# Effect of Risk of Bias on the Effect Size of Meta-Analytic Estimates in Randomized Controlled Trials in Periodontology and Implant Dentistry

**DOI:** 10.1371/journal.pone.0139030

**Published:** 2015-09-30

**Authors:** Clovis Mariano Faggion, Yun-Chun Wu, Moritz Scheidgen, Yu-Kang Tu

**Affiliations:** 1 Department of Periodontology, Faculty of Dentistry, University of Münster, Münster, Germany; 2 Institute of Epidemiology and Preventive Medicine, College of Public Health, National Taiwan University, Taipei, Taiwan; The James Cook University Hospital, UNITED KINGDOM

## Abstract

**Background:**

Risk of bias (ROB) may threaten the internal validity of a clinical trial by distorting the magnitude of treatment effect estimates, although some conflicting information on this assumption exists.

**Objective:**

The objective of this study was evaluate the effect of ROB on the magnitude of treatment effect estimates in randomized controlled trials (RCTs) in periodontology and implant dentistry.

**Methods:**

A search for Cochrane systematic reviews (SRs), including meta-analyses of RCTs published in periodontology and implant dentistry fields, was performed in the Cochrane Library in September 2014. Random-effect meta-analyses were performed by grouping RCTs with different levels of ROBs in three domains (sequence generation, allocation concealment, and blinding of outcome assessment). To increase power and precision, only SRs with meta-analyses including at least 10 RCTs were included. Meta-regression was performed to investigate the association between ROB characteristics and the magnitudes of intervention effects in the meta-analyses.

**Results:**

Of the 24 initially screened SRs, 21 SRs were excluded because they did not include at least 10 RCTs in the meta-analyses. Three SRs (two from periodontology field) generated information for conducting 27 meta-analyses. Meta-regression did not reveal significant differences in the relationship of the ROB level with the size of treatment effect estimates, although a trend for inflated estimates was observed in domains with unclear ROBs.

**Conclusion:**

In this sample of RCTs, high and (mainly) unclear risks of selection and detection biases did not seem to influence the size of treatment effect estimates, although several confounders might have influenced the strength of the association.

## Introduction

Risk of bias (ROB) is an important issue to consider when appraising studies. Generally, the higher the ROB of a study, the less confidence there will be that the results are valid. For example, a meta-analysis formed by randomized clinical trials (RCTs) with low ROB will probably generate stronger evidence to support clinical decision-making than a meta-analysis formed by RCTs with high or unclear ROB. Levels of ROB may interfere with the treatment effect estimates by inflating or reducing the real values. For example, studies with high ROB have been found to generate exaggerated treatment effect estimates [1]. Thus, there is a general consensus that authors of all studies should report all of the measures necessary to produce a study with a low ROB [1, 2].

The Cochrane Collaboration has developed a methodology to evaluate the ROBs of RCTs in different domains [3]. Among other domains, using an adequate randomization process and masking the people involved in the study are important steps to minimize selection and performance/detection biases, respectively. A lack of allocation concealment, an important component of the randomization process, has been demonstrated to inflate treatment estimates in some medical fields [4]. However, attempts to evaluate the effects of different levels of bias on the magnitude of treatment effect estimates have not been performed in periodontology and implant dentistry. Therefore, the objective of this study was to evaluate, in three domains, the influence of ROB on the effect size of meta-analytic estimates in systematic reviews (SRs) of RCTs published in periodontology and implant dentistry.

## Materials and Methods

### Eligibility criteria

To be included in this study, an article should be a SR of RCTs in the fields of periodontology and implant dentistry and published in the Cochrane Database of Systematic Reviews. Excluded from the analysis were SRs published in paper-based journals, SRs including RCTs with noninterventional purposes (e.g., no therapy-related outcome), and SRs including studies with non-RCT designs. Homogeneity of comparisons should allow a meta-analysis to be conducted of the RCTs included in the SR. Therefore, SRs without a meta-analysis were excluded.

### Literature search

**Fig 1:**
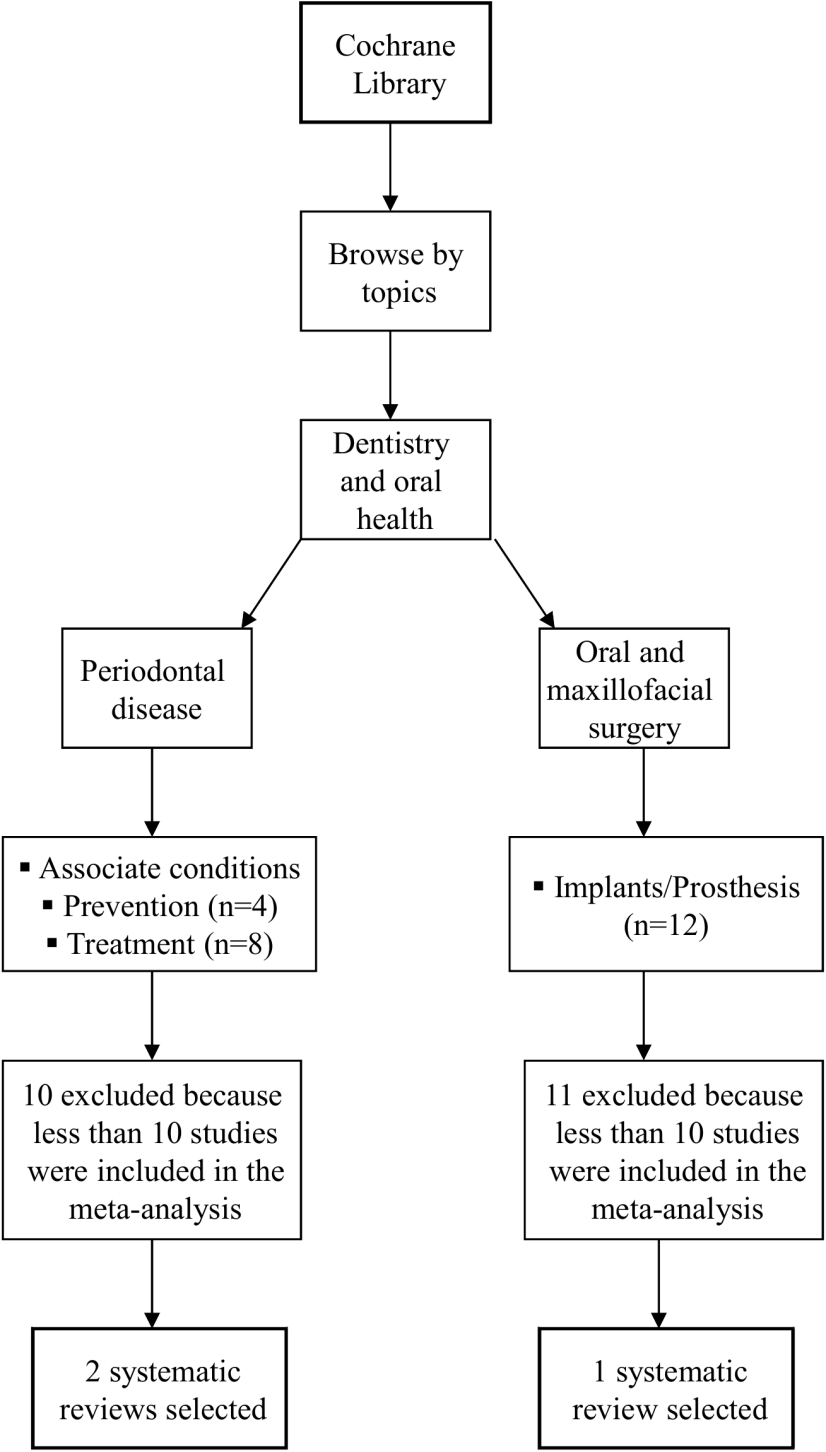
Search for systematic reviews in the Cochrane Library of systematic reviews.

From 15 to 18 September 2014, a literature search was performed in the Cochrane Database of Systematic Reviews for Cochrane SRs on interventions in the fields of periodontology and implant dentistry. Searches were performed directly in the Cochrane Library homepage, by using the “browse by topics” option (Fig 1). Searches were performed independently and in duplicate by two authors (CMF and MS). Any disagreements in SR selection were resolved by discussion until consensus was achieved.

### Rationale for assessment and data extraction

RCTs published in the included SRs were further selected for assessment. SRs from the Cochrane Collaboration were chosen because they use similar approaches for assessing ROB in the primary studies. Furthermore, Cochrane SRs are considered to be of high methodological quality [5, 6], and they may provide the best available evidence possible.

Information about ROB in three domains—namely, sequence generation, allocation concealment, and blinding of outcome assessment—was retrieved directly from the SR reports. Each domain was classified as having a high, low, or unclear ROB by the original authors. No attempt was made to re-evaluate the bias of RCTs included in the SRs. Intervention effects were measured as continuous variables (e.g., mean pocket probing depth and mean clinical attachment level changes) and binary variables (e.g., tooth/implant survival [yes/no] and prosthetic failure [yes/no]).

Meta-analyses of trials with different ROBs within each selected SR were performed and compared. For example, in a hypothetical SR comparing guided tissue regeneration to open flap debridement, treatment effect estimates could be compared between RCTs with different levels of ROB in the three domains. When more than one comparison was made, the treatment estimates were descriptively reported. To increase the power and precision of the assessments, at least 10 RCTs [7, 8] should be included in the meta-analysis, and different ROBs should be included in at least one of the three assessed domains. In addition, to evaluate how much (in percentage) the size of treatment effect estimates differed between groups with different ROBs, a threshold of at least five RCTs in each ROB group was used. Trials included in the SR but not in the meta-analysis were not assessed.

### Statistical assessment

Random-effects pair-wise meta-analyses were performed by grouping RCTs with low, high, and unclear ROBs. Meta-regression analyses [7, 9] were undertaken to investigate differences in treatment outcomes between trials with high/unclear ROBs and those with low ROB in the aforementioned three domains of bias. The impact of any of the domains of bias was then investigated.

P-values of heterogeneity between ROB groups were obtained by comparing the statistic with the *t* distribution. Forest plots were stratified by high, low, and unclear ROB. All analyses were undertaken by using the statistical software package Stata (version 12, StataCorp).

## Results

### Literature search

The search in the Cochrane Database for Systematic Reviews retrieved 24 potential SRs with meta-analyses dealing with subjects related to periodontology and implant dentistry. Twenty-one SRs were excluded after full-text assessment, leaving three SRs for statistical assessment (Fig 1). Excluded SRs are reported in the S1 File.

### Characteristics and risks of bias of included SRs

Two SRs were published in the periodontology field [10, 11] and one in the implant dentistry field [12]. Table 1 reports the characteristics of the included SRs and the number of RCTs in each SR. S1 Table, S2 Table and S3 Table depict the ROB scores for the 107 RCTs reported in the three selected SRs.

**Table 1:** Characteristics of included systematic reviews.

Systematic review	Field	Number of RCTs*	Interventions	Outcomes evaluated
**Esposito et al. 2013**	Implant dentistry	n = 26	Root-form osseointegrated dental implants, having a follow-up of 4 months to 1 year, comparing the same implant type immediately, early or conventionally loaded, occlusally or non-occlusally loaded, or progressively loaded or not	Prosthesis and implant failures and radiographic marginal bone level changes
**Riley and Lamont 2013**	Periodontology	n = 30	Assessment of the effects of triclosan/copolymer containing fluoride toothpastes, compared with fluoride toothpastes, for the long-term control of caries, plaque and gingivitis in children and adults	Primary outcomes (plaque levels measured using any appropriate scale, gingival health measured using any appropriate scale); Secondary outcomes (incidence of periodontitis, caries: a) new incidence, and b) caries increment–change in decayed, missing and filled surfaces (DMFS/dmfs) index, calculus measured using any appropriate scale, adverse effects (e.g. taste disturbance, staining, allergic reaction, etc.), participant-centred outcomes: a) participant-assessed quality of life scores, and b) participant satisfaction with product.
**Yaacob et al. 2014**	Periodontology	n = 51	Unsupervised powered toothbrushing versus manual toothbrushing for oral health in children and adults	Primary outcomes (quantified levels of plaque or gingivitis or both), Secondary outcome measures sought were levels of calculus and staining; dependability and cost of the brush used, including mechanical deterioration; and adverse effects such as hard or soft tissue injury and damage to orthodontic appliances and prostheses.

*RCTs included in the meta-analyses only

### Influence of ROB on the estimates

Fifty-seven meta-analyses of RCTs with different levels of ROB and types of clinical outcomes in different therapeutic approaches are reported in 26 forest plots (S2 File). Most comparisons were between RCTs with low and unclear ROBs. Meta-regression assessment showed that one meta-analytic comparison provided less inflated treatment effect estimates in the subgroup formed by RCTs with low ROB (Table 2). In this assessment, the comparator was formed by a subgroup of RCTs with unclear ROB, and the domain evaluated was blinding of outcome assessment.

**Table 2:** Meta-regression assessment of the influence of different levels of risk of bias on the treatment effect estimates. (1): Sequence generation; (2): Allocation concealment; (3) Blinding of outcome assessment; (4) Sequence generation OR Allocation concealment; (5) Sequence generation OR Blinding of outcome assessment; (6) Allocation concealment OR Blinding of outcome assessment; (7) Sequence generation OR Allocation concealment ORBlinding of outcome assessment. NA = not available

Study	Comparison/ Outcome	Risk of bias				
		(1)	(2)	(3)	(4)	(5)
**Riley and Lamont 2013 (Peridontology)**	Plaque at 6 to 7 months vs. Baseline prophylaxis					
	**(A-1)** Quigley-Hein Plaque Index	0.665	0.123	NA	0.123	0.665
	Plaque Severity Index versus Baseline prophylaxis					
	**(A-2)** Plaque Severity Index	NA	NA	NA	NA	NA
	Gingivitis at 6 to 9 month versus. Baseline prophylaxis					
	**(A-3)** Löe-Silness Gingival Index	0.669	0.244	NA	0.244	0.669
	Gingivitis at 6 to 7 month versus Baseline prophylaxis					
	**(A-4)** Gingivitis Severity Index	0.055	0.055	NA	0.055	0.055
**Yaacob et al. 2014 (Periodontology)**	All powered toothbrushes versus manual toothbrushes					
	**(A-5)** Plaque scores at 1 to 3 months at all sites	0.535	0.405	0.850	0.400	0.535
	Gingival scores at 1 to 3 months at all sites					
	**(A-6)** Loe and Silness	0.846	0.459	0.053	0.457	0.846
	**(A-7)** Plaque scores at >3 months	0.313	0.371	0.439	0.371	0.313
	Rotation oscillation powered toothbrushes versus manual toothbrushes					
	**(A-8)** Plaque scores at 1 to 3 month at all sites	0.471	0.375	NA	0.375	0.471
	**(A-9)** Gingival scores at 1 to 3 months at all sites	0.069	0.244	0.029*	0.277	0.069
**Esposito et al. 2013 (Implant Dentistry)**	Immediate versus conventional loading					
	**(B-1)** Implant failure	0.825	0.893	0.971	0.893	0.881

* p-value<0.05

The other 56 meta-analytic comparisons did not show any significant difference between the effects of low, high, or unclear ROB on the magnitude of treatment effect estimates. However, some potential trends in the influence of bias level on the estimates were observed in seven evaluations with at least five RCTs in each compared group. In these seven meta-analyses, domains with unclear ROB generated more inflated treatment effect estimates, in a range of 21% to 193% (median 39%, Table 3).

**Table 3:** Magnitude of the influence (in percentage) of different levels of risk of bias on the treatment effect estimates (only comparisons with at least 5 studies in each ROB group).

Study	Treatments	Domains	Domains comparison	Outcome measure	Effect	P Value
**Yaacob et al. 2014**	All powered toothbrushes versus manual toothbrushes	Sequence generation and sequence generation or blinding of outcome assessment	Unclear versus low ROB	Plaque scores at 1 to 3 months at all sites	35.00% higher in unclear ROB	0.535
**Yaacob et al. 2014**	All powered toothbrushes versus manual toothbrushes	Allocation concealment and allocation concealment or blinding of outcome assessment	Unclear versus low ROB	Plaque scores at 1 to 3 months at all sites	142.00% higher in unclear ROB	0.405
**Yaacob et al. 2014**	All powered toothbrushes versus manual toothbrushes	Sequence generation and sequence generation or blinding of outcome assessment	Unclear versus low ROB	Löe and Silness index	21.00% higher in unclear ROB	0.846
**Yaacob et al. 2014**	All powered toothbrushes versus manual toothbrushes	Allocation concealment and allocation concealment or blinding of outcome assessment	Unclear versus low ROB	Löe and Silness index	39.00% higher in unclear ROB	0.459
**Yaacob et al. 2014**	All powered toothbrushes versus manual toothbrushes	Blinding of outcome assessment	Unclear versus low ROB	Löe and Silness index	131.00% higher in unclear ROB	0.053
**Yaacob et al. 2014**	Rotation oscillation powered toothbrushes versus manual toothbrushes	Sequence generation and sequence generation or blinding of outcome assessment	Unclear versus low ROB	Plaque scores at 1 to 3 months at all sites	24.00% higher in unclear ROB	0.471
**Yaacob et al. 2014**	Rotation oscillation powered toothbrushes versus manual toothbrushes	Sequence generation and sequence generation or blinding of outcome assessment	Unclear versus low ROB	Gingival scores at 1 to 3 months at all sites	193.00% higher in unclear ROB	0.069

## Discussion

In this sample of SRs, there was no difference in the influence of different levels of ROB on the size of treatment effect estimates, although some trends for more exaggerated estimates were observed in domains with unclear ROB. These trends were based on point estimates, without accounting for the uncertainty in the point estimates. Overlapping of the confidence intervals of the estimates represents the nonsignificant results in an inferential perspective. However, the trend for more exaggerated estimates confirms the results of previous studies that evaluated the influence of different levels of ROB on the treatment effect estimates [13, 14].

We only evaluated the influences of three types of bias (selection, performance, and detection biases) on treatment effect estimates. Too few RCTs with heterogeneous levels of bias (e.g., high versus low ROB; S1 Table, S2 Table and S3 Table) in other domains were available for meta-analyses and meta-regressions to be performed. To increase the power and precision of the assessment, our inclusion criteria stipulated that only original meta-analyses (performed by Cochrane authors) including more than 10 RCTs be included. We made no attempt to increase the number of RCTs to be compared by using alternative approaches, such as including RCTs that were excluded by Cochrane authors. Such alternative approaches would add bias to the study, due to the heterogeneity among these RCTs. For example, the authors of one SR [11] excluded some reports of RCTs due to their short follow-up period. If the excluded RCTs were to be compared with the included RCTs, then the results would be misleading because treatment effect estimates with different follow-up periods will very likely be heterogeneous. Therefore, we only included and compared domains of RCTs that met strict eligibility criteria, to allow the most homogeneous comparison possible and to avoid any bias or confounders that would hinder a reasonable conclusion to be made.

The level of ROB used in the meta-analytic assessments was that reported by the authors of the SRs. We did not make any attempt to reclassify the ROBs in the evaluated domains. Nevertheless, we believe that using the original authors’ evaluations of ROB is the most realistic approach because it represents the data used by end-users of SRs.

The most frequently reported levels of ROB were “low” and “unclear”. Low ROB means that the authors of the SR are relatively confident that the specific domain assessed will not pose any threat to the internal validity of the study. In an “unclear” ROB case, the authors do not have sufficient information to assign the ROB level as low or high. Therefore, some of the domains classified as unclear may, in fact, be domains with low ROB. Clarifying this issue is not an easy task. The Cochrane Collaboration recommends that the authors of the primary studies included in the SR be contacted to obtain more information for the correct judgement of the ROB level [3]. Whether these extra efforts for clarifying the data are enough to provide accurate information remains unknown. Authors of the SRs included in the present work reported they contacted authors of primary studies to clarify issues, although it was not explicitly reported whether this contact also involved the ROB evaluation. We did not contact the authors of RCTs included to not deviate from our original protocol.

In the present study, one selected SR [11] fulfilled the predefined threshold of five trials in each ROB group in order to evaluate how much the size of treatment effect estimates differed (in percentage) between domains of low and unclear ROBs. Meta-regression assessment demonstrated that differences in treatment outcomes between trials with high/unclear ROBs and those with low ROB were not significant, although there was a trend for more inflated estimates in domains with unclear ROB. In fact, two of the seven comparisons barely reached the significant level, although most of these comparisons represented substantial differences in percentage.

There are conflicting results in the literature regarding the influence of different levels of bias on the magnitude of treatment effect estimates. In two studies [4, 13], treatment effect estimates were inflated by 30–50% when procedures to reduce ROB were not adequately addressed in some domains. In contrast, another study did not find any significant influence of certain quality measures on the size of treatment effect estimates [15]. Potential explanations for this variability of results include the lack of uniformity in defining levels of bias or quality and the heterogeneity of clinical fields evaluated, which may include domains with different susceptibilities to bias [15]. Our stricter threshold for including trials in the meta-regression might be one potential reason that we did not detect significant differences between trials with high/unclear ROBs.

One may argue that there were not enough RCTs included in the meta-regression assessment to have sufficient power to detect any difference. We followed the previously reported suggestion that, to reduce the chance of type II error, at least 10 studies should be included in the meta-regression assessment [7, 8]. Some researchers have suggested using a minimum of six studies in the meta-regression model for a continuous study level variable [16]. However, raising the threshold for the minimal number of trials will likely provide more accurate and meaningful results by increasing precision. Furthermore, when reporting potential trends in the influence of bias level on the estimates, we included meta-regression evaluations that had at least five RCTs in each ROB level group (i.e., low, unclear, or high). Nevertheless, even with these efforts, we cannot rule out the potential lack of power of the evaluations, and the results should be considered with caution.

Two approaches can be taken to test the impact of a categorical variable on the results of a meta-analysis. One approach is to undertake a stratified or subgroup meta-analysis, and the other is to undertake meta-regression analysis by including the categorical variable as an explanatory variable. We used the latter approach in the present study; however, both approaches should yield very similar results.

## Conclusion

Although no statistical difference was observed, the results showed a trend for more inflated estimates in trials with domains of unclear ROB. The present results may contribute to the understanding of the influence of ROB on treatment effect estimates across medical disciplines.

## Supporting Information

S1 File List of excluded systematic reviews. (DOCX)

S2 File Forest plots of meta-analyses comparing studies with different types of risk of bias. (PDF)

S1 Table Risk of bias summary of RCTs included in Esposito et al. 2013. (DOCX)

S2 Table Risk of bias summary of RCTs included in Riley and Lamont 2013. (DOCX)

S3 Table Risk of bias summary of RCTs included in Yaacob et al. 2013. (DOCX)
