# Correction: Effect of Risk of Bias on the Effect Size of Meta-Analytic Estimates in Randomized Controlled Trials in Periodontology and Implant Dentistry

**DOI:** 10.1371/journal.pone.0142233

**Published:** 2015-11-10

**Authors:** Clovis Mariano Faggion, Yun-Chun Wu, Moritz Scheidgen, Yu-Kang Tu

There is an error in Table 2. Columns 6 and 7 are missing. Please see the corrected Table 2 here.

**Table 2:** Meta-regression assessment of the influence of different levels of risk of bias on the treatment effect estimates. (1): Sequence generation; (2): Allocation concealment; (3) Blinding of outcome assessment; (4) Sequence generation OR Allocation concealment; (5) Sequence generation OR Blinding of outcome assessment; (6) Allocation concealment OR Blinding of outcome assessment; (7) Sequence generation OR Allocation concealment OR Blinding of outcome assessment. NA = not available

Study	Comparison/ Outcome	Risk of bias						
		(1)	(2)	(3)	(4)	(5)	(6)	(7)
**Riley and Lamont 2013 (Peridontology)**	Plaque at 6 to 7 months vs. Baseline prophylaxis							
	**(A-1)** Quigley-Hein Plaque Index	0.665	0.123	NA	0.123	0.665	0.123	0.123
	Plaque Severity Index versus Baseline prophylaxis							
	**(A-2)** Plaque Severity Index	NA	NA	NA	NA	NA	NA	NA
	Gingivitis at 6 to 9 month versus. Baseline prophylaxis							
	**(A-3)** Löe-Silness Gingival Index	0.669	0.244	NA	0.244	0.669	0.244	0.244
	Gingivitis at 6 to 7 month versus Baseline prophylaxis							
	**(A-4)** Gingivitis Severity Index	0.055	0.055	NA	0.055	0.055	0.055	0.055
**Yaacob et al. 2014 (Periodontology)**	All powered toothbrushes versus manual toothbrushes							
	**(A-5)** Plaque scores at 1 to 3 months at all sites	0.535	0.405	0.850	0.400	0.535	0.405	0.400
	Gingival scores at 1 to 3 months at all sites							
	**(A-6)** Loe and Silness	0.846	0.459	0.053	0.457	0.846	0.459	0.457
	**(A-7)** Plaque scores at >3 months	0.313	0.371	0.439	0.371	0.313	0.371	0.371
	Rotation oscillation powered toothbrushes versus manual toothbrushes							
	**(A-8)** Plaque scores at 1 to 3 month at all sites	0.471	0.375	NA	0.375	0.471	0.375	0.375
	**(A-9)** Gingival scores at 1 to 3 months at all sites	0.069	0.244	0.029*	0.277	0.069	0.244	0.277
**Esposito et al. 2013 (Implant Dentistry)**	Immediate versus conventional loading							
	**(B-1)** Implant failure	0.825	0.893	0.971	0.893	0.881	NA	NA

* p-value<0.05
