# Supplementary material for: Effect of Risk of Bias on the Effect Size of Meta-Analytic Estimates in Randomized Controlled Trials in Periodontology and Implant Dentistry
Source: PLoS One. 2015 Sep 30;10(9):e0139030. doi: 10.1371/journal.pone.0139030 (PMC4589402; doi:10.1371/journal.pone.0139030)
Supplement: S1 File — (DOCX) [file pone.0139030.s001.docx]

**Supporting Information**

**S1 file: List of excluded systematic reviews**

Coulthard P, Esposito M, Worthington HV, Jokstad A. 2002. Interventions for replacing missing teeth: preprosthetic surgery versus dental implants. Cochrane Database of Systematic Reviews, Issue 4. Art. No.: CD003604.

Deacon SA, GlennyAM, Deery C, Robinson PG, Heanue M, Walmsley AD, Shaw WC. 2010. Different powered toothbrushes for plaque control and gingival health. Cochrane Database of Systematic Reviews, Issue 12. Art. No.: CD004971.

Eberhard J, Jepsen S, Jervøe-Storm PM, Needleman I, Worthington HV. 2008. Full-mouth disinfection for the treatment of adult chronic periodontitis. Cochrane Database of Systematic Reviews 2008, Issue 1. Art. No.: CD004622.

Esposito M, Grusovin MG, Papanikolaou N, Coulthard P, Worthington HV. 2009a. Enamel matrix derivative (Emdogain®) for periodontal tissue regeneration in intrabony defects. Cochrane Database of Systematic Reviews, Issue 4. Art. No.: CD003875.

Esposito M, Grusovin MG, Chew YS, Coulthard P, Worthington HV. 2009b. Interventions for replacing missing teeth: 1- versus 2-stage implant placement. Cochrane Database of Systematic Reviews, Issue 3. Art. No.: CD006698.

Esposito M, Grusovin MG, Felice P, Karatzopoulos G, Worthington HV, Coulthard P. 2009c. Interventions for replacing missing teeth: horizontal and vertical bone augmentation techniques for dental implant treatment. Cochrane Database of Systematic Reviews, Issue 4. Art. No.: CD003607.

Esposito M, Grusovin MG, Polyzos IP, Felice P, Worthington HV. 2010. Interventions for replacing missing teeth: dental implants in fresh extraction sockets (immediate, immediate-delayed and delayed implants). Cochrane Database of Systematic Reviews, Issue 9. Art. No.: CD005968.

Esposito M, Maghaireh H, Grusovin MG, Ziounas I, Worthington HV. 2012a. Interventions for replacing missing teeth: management of soft tissues for dental implants. Cochrane Database of Systematic Reviews, Issue 2. Art. No.: CD006697.

Esposito M, Grusovin MG, Worthington HV. 2012b. Interventions for replacing missing teeth: treatment of peri-implantitis. Cochrane Database of Systematic Reviews, Issue 1. Art. No.: CD004970.

Esposito M, Worthington HV. 2013a. Interventions for replacing missing teeth: dental implants in zygomatic bone for the rehabilitation of the severely deficient edentulous maxilla. Cochrane Database of Systematic Reviews, Issue 9. Art. No.: CD004151.

Esposito M, Worthington HV. 2013b. Interventions for replacing missing teeth: hyperbaric oxygen therapy for irradiated patients who require dental implants. Cochrane Database of Systematic Reviews, Issue 9. Art. No.: CD003603.

Esposito M, Grusovin MG, Worthington HV. 2013. Interventions for replacing missing teeth: antibiotics at dental implant placement to prevent complications. Cochrane Database of Systematic Reviews, Issue 7. Art. No.: CD004152.

Esposito M, Ardebili Y, Worthington HV. 2014. Interventions for replacing missing teeth: different types of dental implants. Cochrane Database of Systematic Reviews, Issue 7. Art. No.: CD003815.

Grusovin MG, Coulthard P, Worthington HV, George P, Esposito M. 2010. Interventions for replacing missing teeth: maintaining and recovering soft tissue health around dental implants. Cochrane Database of Systematic Reviews, Issue 8. Art. No.: CD003069.

Needleman I, Worthington HV, Giedrys-Leeper E, Tucker R. 2006.Guided tissue regeneration for periodontal infra-bony defects. Cochrane Database of Systematic Reviews, Issue 2. Art. No.: CD001724.

Poklepovic T, Worthington HV, Johnson TM, Sambunjak D, Imai P, Clarkson JE, Tugwell P. 2013. Interdental brushing for the prevention and control of periodontal diseases and dental caries in adults. Cochrane Database of Systematic Reviews, Issue 12. Art. No.: CD009857.

Renz A, Ide M, Newton T, Robinson P, Smith D. 2007.Psychological interventions to improve adherence to oral hygiene instructions in adults with periodontal diseases. Cochrane Database of Systematic Reviews, Issue 2. Art. No.: CD005097.

Sambunjak D, Nickerson JW, Poklepovic T, Johnson TM, Imai P, Tugwell P, Worthington HV. 2011. Flossing for the management of periodontal diseases and dental caries in adults. Cochrane Database of Systematic Reviews, Issue 12. Art. No.: CD008829.

Simpson TC, Needleman I, Wild SH, Moles DR, Mills EJ. 2010. Treatment of periodontal disease for glycaemic control in people with diabetes. Cochrane Database of Systematic Reviews, Issue 5. Art. No.: CD004714.

Weston P, Yaziz YA, Moles DR, Needleman I. Occlusal interventions for periodontitis in adults. Cochrane Database of Systematic Reviews 2008, Issue 3. Art. No.: CD004968.

Worthington HV, Clarkson JE, Bryan G, Beirne PV. 2013. Routine scale and polish for periodontal health in adults. Cochrane Database of Systematic Reviews, Issue 11. Art. No.: CD004625.
