# Supplementary material for: Effect of Risk of Bias on the Effect Size of Meta-Analytic Estimates in Randomized Controlled Trials in Periodontology and Implant Dentistry
Source: PLoS One. 2015 Sep 30;10(9):e0139030. doi: 10.1371/journal.pone.0139030 (PMC4589402; doi:10.1371/journal.pone.0139030)
Supplement: S2 File — (PDF) [file pone.0139030.s002.pdf]

Pair-wise meta-analyses comparing groups with different levels of ROB

Riley and Lamont 2013

Figure A-1 (1, 5)

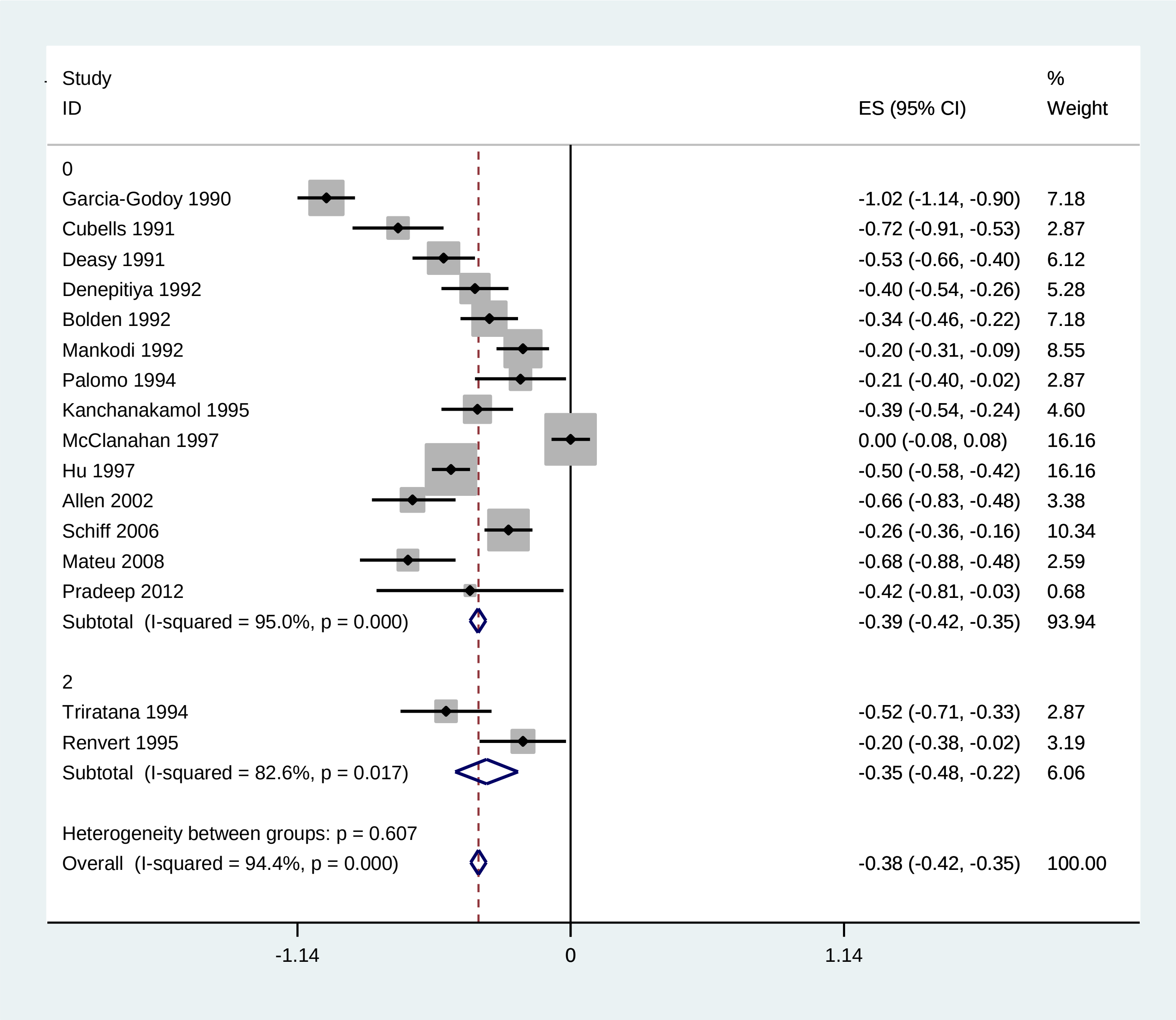

Figure A-1 (2, 4, 6, 7)

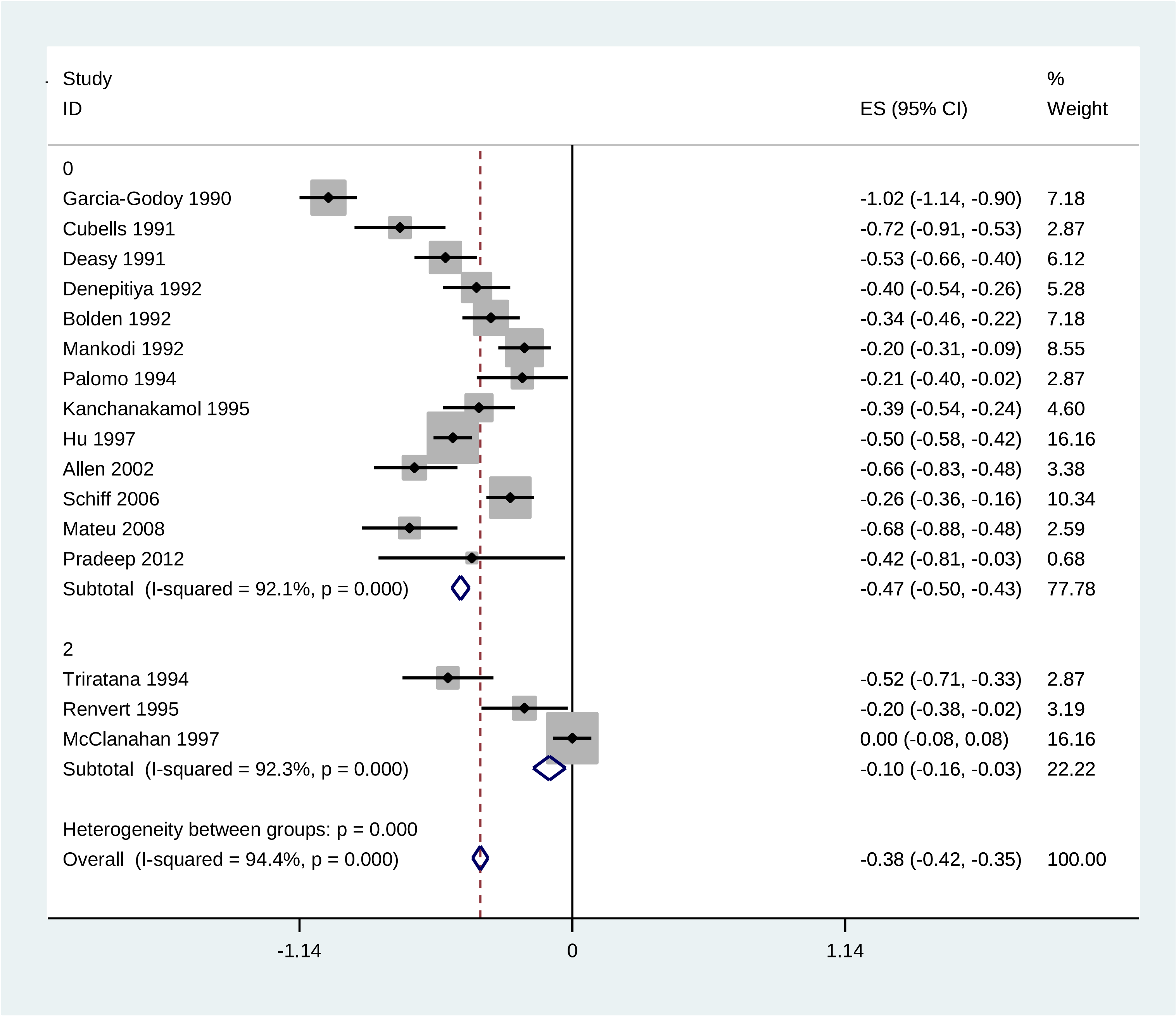

A-3 (1, 5)

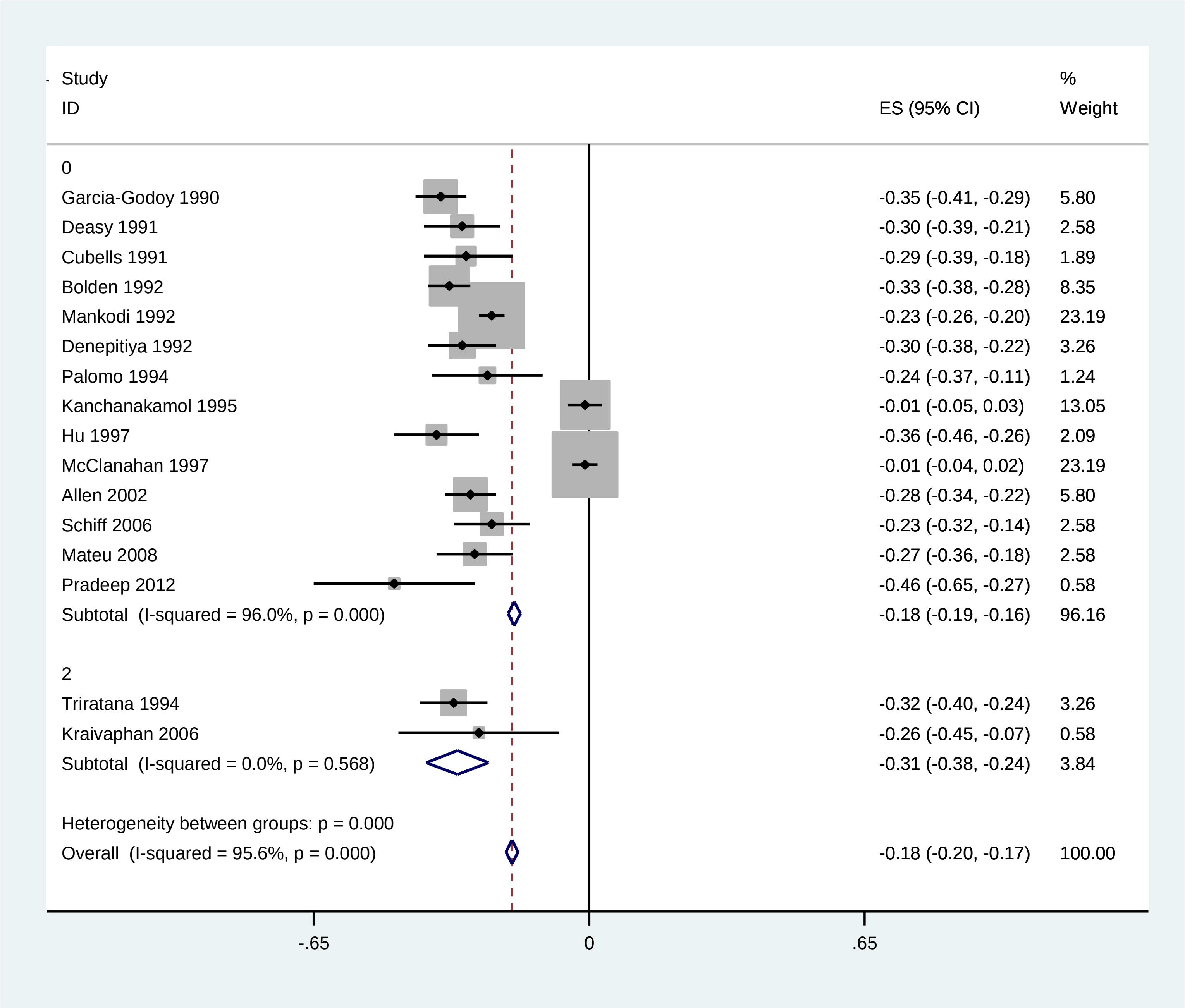

Study
ID
ES (95% CI)
%
Weight
0
Garcia-Godoy 1990 -0.35 (-0.41, -0.29) 5.80
Deasy 1991 -0.30 (-0.39, -0.21) 2.58
Cubells 1991 -0.29 (-0.39, -0.18) 1.89
Bolden 1992 -0.33 (-0.38, -0.28) 8.35
Mankodi 1992 -0.23 (-0.26, -0.20) 23.19
Denepitiya 1992 -0.30 (-0.38, -0.22) 3.26
Palomo 1994 -0.24 (-0.37, -0.11) 1.24
Kanchanakamol 1995 -0.01 (-0.05, 0.03) 13.05
Hu 1997 -0.36 (-0.46, -0.26) 2.09
McClanahan 1997 -0.01 (-0.04, 0.02) 23.19
Allen 2002 -0.28 (-0.34, -0.22) 5.80
Schiff 2006 -0.23 (-0.32, -0.14) 2.58
Mateu 2008 -0.27 (-0.36, -0.18) 2.58
Pradeep 2012 -0.46 (-0.65, -0.27) 0.58
Subtotal (I-squared = 96.0%, p = 0.000) -0.18 (-0.19, -0.16) 96.16
2
Triratana 1994 -0.32 (-0.40, -0.24) 3.26
Kraivaphan 2006 -0.26 (-0.45, -0.07) 0.58
Subtotal (I-squared = 0.0%, p = 0.568) -0.31 (-0.38, -0.24) 3.84
Heterogeneity between groups: p = 0.000
Overall (I-squared = 95.6%, p = 0.000) -0.18 (-0.20, -0.17) 100.00
-.65
0
.65

A-3 (2,4,6,7)

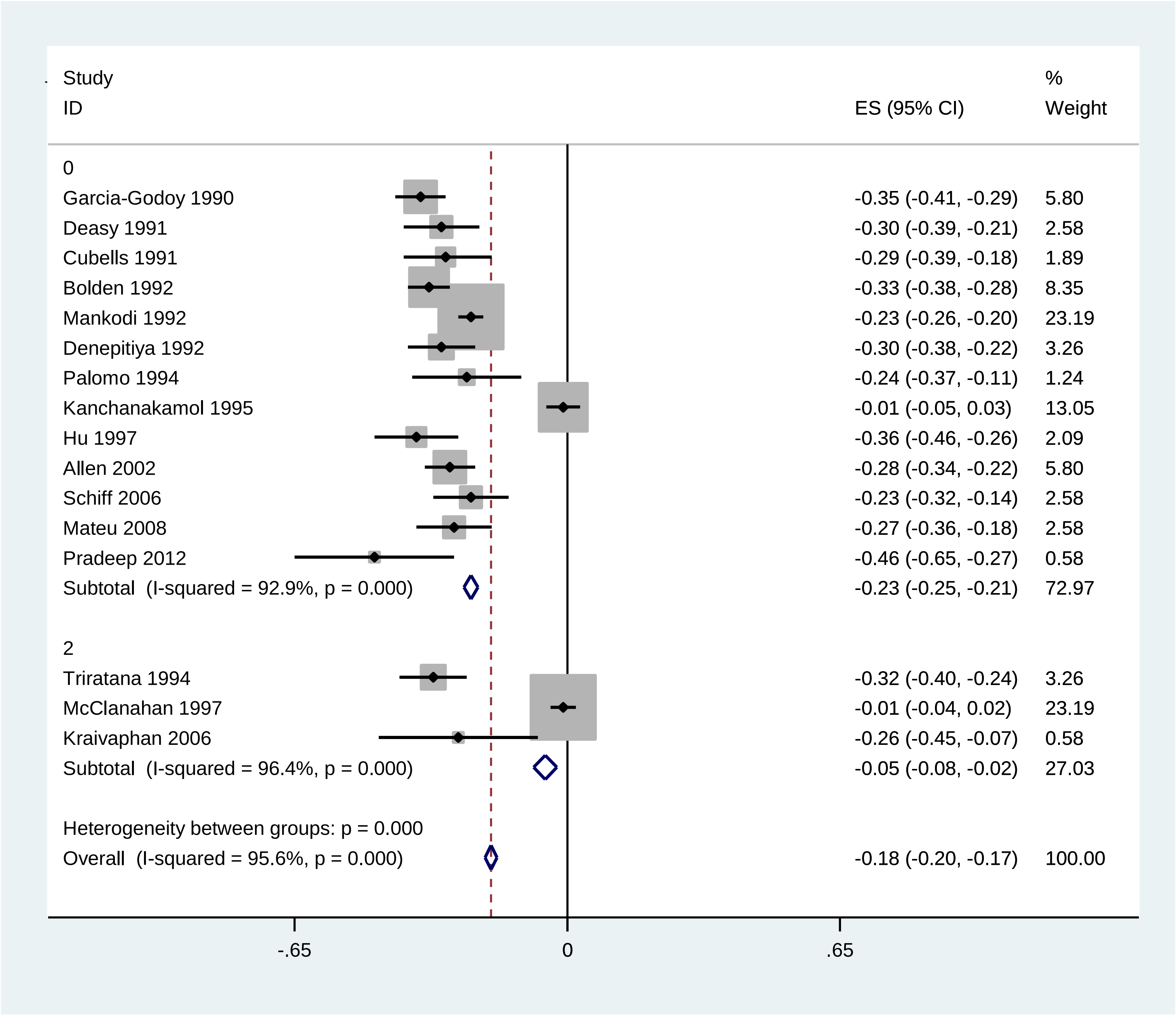
Study
ID
ES (95% CI)
%
Weight
0
Garcia-Godoy 1990 -0.35 (-0.41, -0.29) 5.80
Deasy 1991 -0.30 (-0.39, -0.21) 2.58
Cubells 1991 -0.29 (-0.39, -0.18) 1.89
Bolden 1992 -0.33 (-0.38, -0.28) 8.35
Mankodi 1992 -0.23 (-0.26, -0.20) 23.19
Denepitiya 1992 -0.30 (-0.38, -0.22) 3.26
Palomo 1994 -0.24 (-0.37, -0.11) 1.24
Kanchanakamol 1995 -0.01 (-0.05, 0.03) 13.05
Hu 1997 -0.36 (-0.46, -0.26) 2.09
Allen 2002 -0.28 (-0.34, -0.22) 5.80
Schiff 2006 -0.23 (-0.32, -0.14) 2.58
Mateu 2008 -0.27 (-0.36, -0.18) 2.58
Pradeep 2012 -0.46 (-0.65, -0.27) 0.58
Subtotal (I-squared = 92.9%, p = 0.000) -0.23 (-0.25, -0.21) 72.97
2
Triratana 1994 -0.32 (-0.40, -0.24) 3.26
McClanahan 1997 -0.01 (-0.04, 0.02) 23.19
Kraivaphan 2006 -0.26 (-0.45, -0.07) 0.58
Subtotal (I-squared = 96.4%, p = 0.000) -0.05 (-0.08, -0.02) 27.03
Heterogeneity between groups: p = 0.000
Overall (I-squared = 95.6%, p = 0.000) -0.18 (-0.20, -0.17) 100.00
-.65
0
.65

A-4 (1, 2, 4-7)

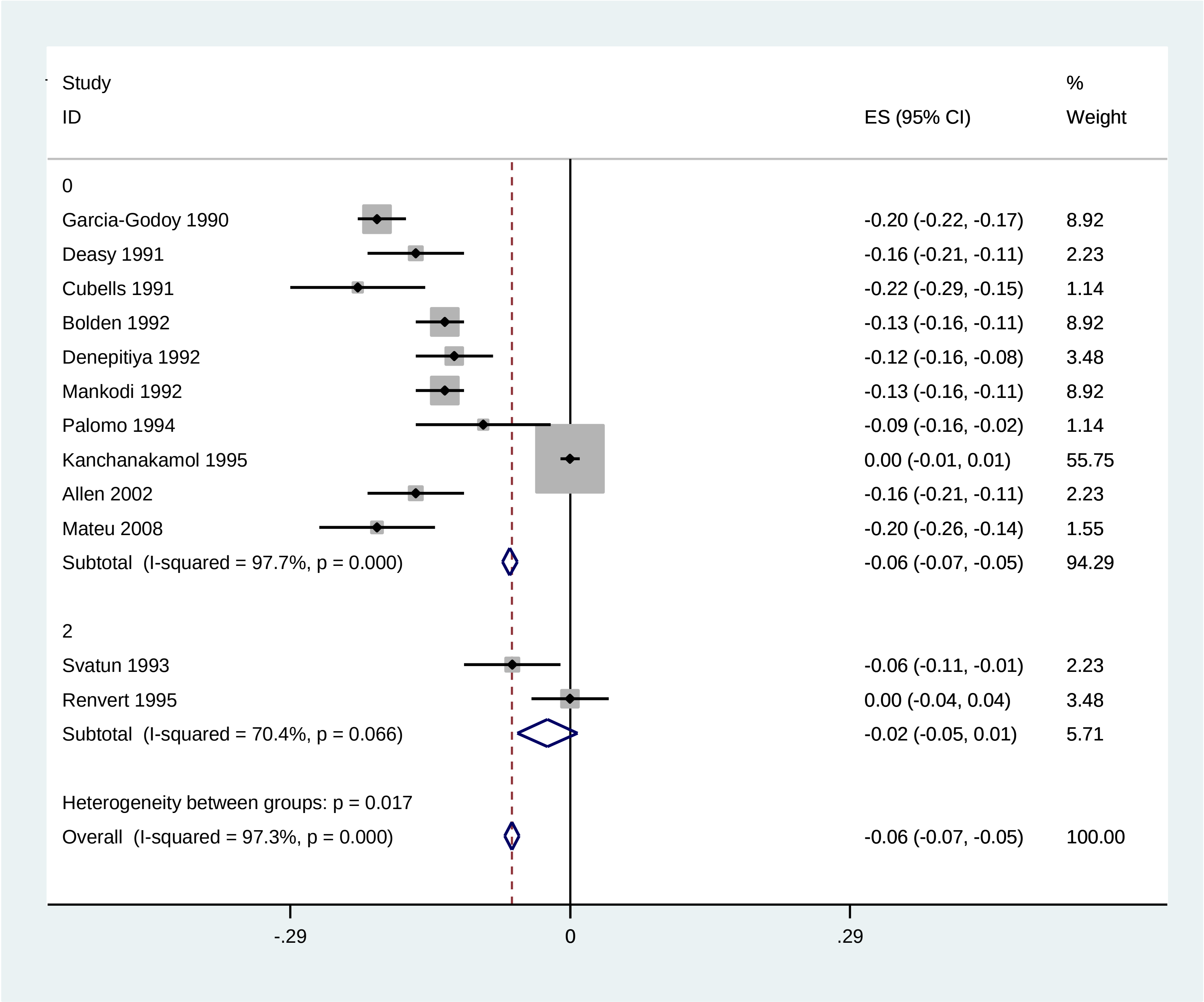
Study
ID
ES (95% CI)
%
Weight
0
Garcia-Godoy 1990 -0.20 (-0.22, -0.17) 8.92
Deasy 1991 -0.16 (-0.21, -0.11) 2.23
Cubells 1991 -0.22 (-0.29, -0.15) 1.14
Bolden 1992 -0.13 (-0.16, -0.11) 8.92
Denepitiya 1992 -0.12 (-0.16, -0.08) 3.48
Mankodi 1992 -0.13 (-0.16, -0.11) 8.92
Palomo 1994 -0.09 (-0.16, -0.02) 1.14
Kanchanakamol 1995 0.00 (-0.01, 0.01) 55.75
Allen 2002 -0.16 (-0.21, -0.11) 2.23
Mateu 2008 -0.20 (-0.26, -0.14) 1.55
Subtotal (I-squared = 97.7%, p = 0.000) -0.06 (-0.07, -0.05) 94.29
2
Svatun 1993 -0.06 (-0.11, -0.01) 2.23
Renvert 1995 0.00 (-0.04, 0.04) 3.48
Subtotal (I-squared = 70.4%, p = 0.066) -0.02 (-0.05, 0.01) 5.71
Heterogeneity between groups: p = 0.017
Overall (I-squared = 97.3%, p = 0.000) -0.06 (-0.07, -0.05) 100.00
-.29
0
.29

Yaacob et al. 2014

A-5 (1, 5)

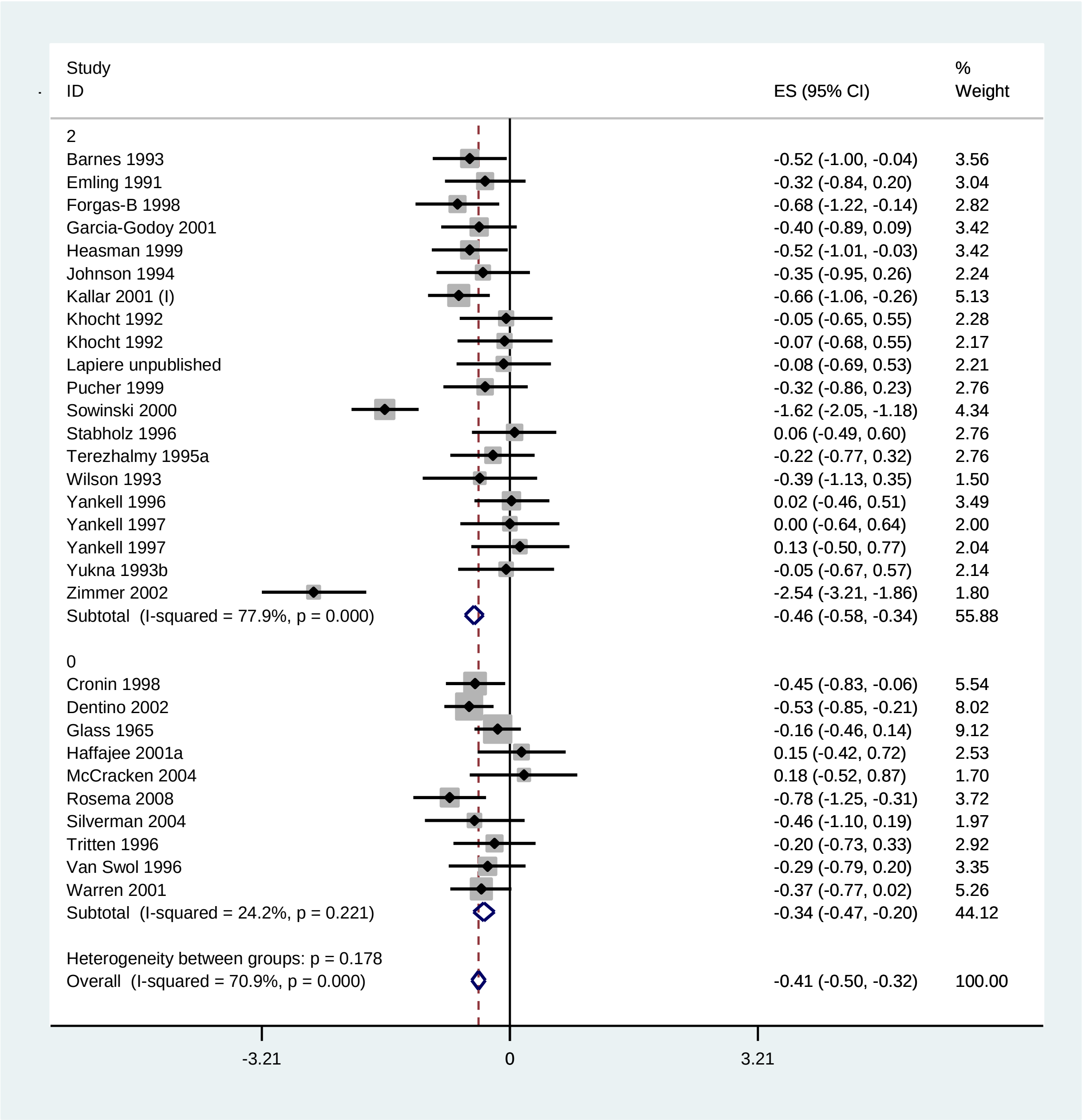

A-5 (2, 6)

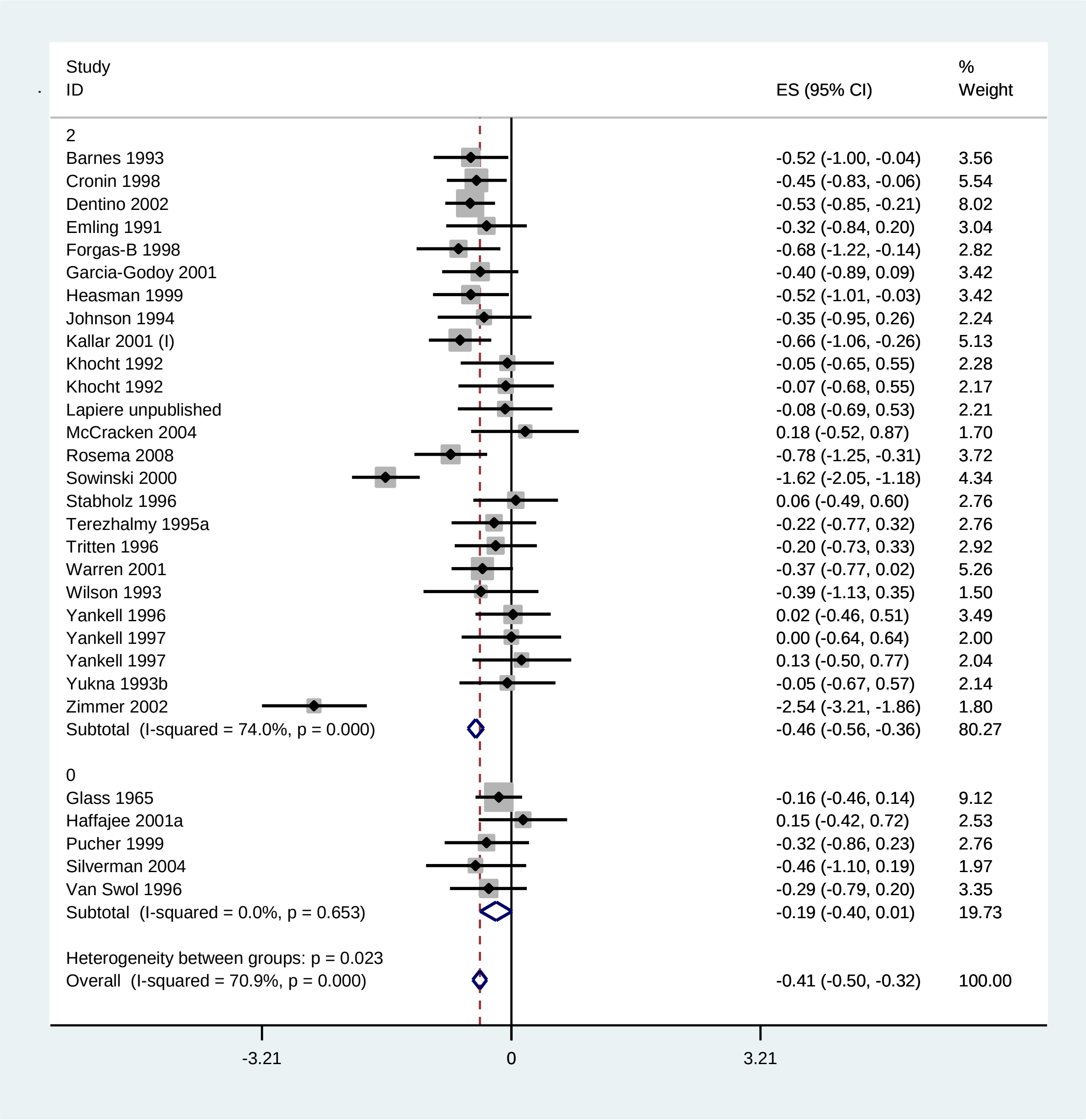
Study
ID
ES (95% CI)
%
Weight
2
Barnes 1993 -0.52 (-1.00, -0.04) 3.56
Cronin 1998 -0.45 (-0.83, -0.06) 5.54
Dentino 2002 -0.53 (-0.85, -0.21) 8.02
Emling 1991 -0.32 (-0.84, 0.20) 3.04
Forgas-B 1998 -0.68 (-1.22, -0.14) 2.82
Garcia-Godoy 2001 -0.40 (-0.89, 0.09) 3.42
Heasman 1999 -0.52 (-1.01, -0.03) 3.42
Johnson 1994 -0.35 (-0.95, 0.26) 2.24
Kallar 2001 (I) -0.66 (-1.06, -0.26) 5.13
Khocht 1992 -0.05 (-0.65, 0.55) 2.28
Khocht 1992 -0.07 (-0.68, 0.55) 2.17
Lapiere unpublished -0.08 (-0.69, 0.53) 2.21
McCracken 2004 0.18 (-0.52, 0.87) 1.70
Rosema 2008 -0.78 (-1.25, -0.31) 3.72
Sowinski 2000 -1.62 (-2.05, -1.18) 4.34
Stabholz 1996 0.06 (-0.49, 0.60) 2.76
Terezhalmy 1995a -0.22 (-0.77, 0.32) 2.76
Tritten 1996 -0.20 (-0.73, 0.33) 2.92
Warren 2001 -0.37 (-0.77, 0.02) 5.26
Wilson 1993 -0.39 (-1.13, 0.35) 1.50
Yankell 1996 0.02 (-0.46, 0.51) 3.49
Yankell 1997 0.00 (-0.64, 0.64) 2.00
Yankell 1997 0.13 (-0.50, 0.77) 2.04
Yukna 1993b -0.05 (-0.67, 0.57) 2.14
Zimmer 2002 -2.54 (-3.21, -1.86) 1.80
Subtotal (I-squared = 74.0%, p = 0.000) -0.46 (-0.56, -0.36) 80.27
0
Glass 1965 -0.16 (-0.46, 0.14) 9.12
Haffajee 2001a 0.15 (-0.42, 0.72) 2.53
Pucher 1999 -0.32 (-0.86, 0.23) 2.76
Silverman 2004 -0.46 (-1.10, 0.19) 1.97
Van Swol 1996 -0.29 (-0.79, 0.20) 3.35
Subtotal (I-squared = 0.0%, p = 0.653) -0.19 (-0.40, 0.01) 19.73
Heterogeneity between groups: p = 0.023
Overall (I-squared = 70.9%, p = 0.000) -0.41 (-0.50, -0.32) 100.00
-3.21
0
3.21

A-5 (3)

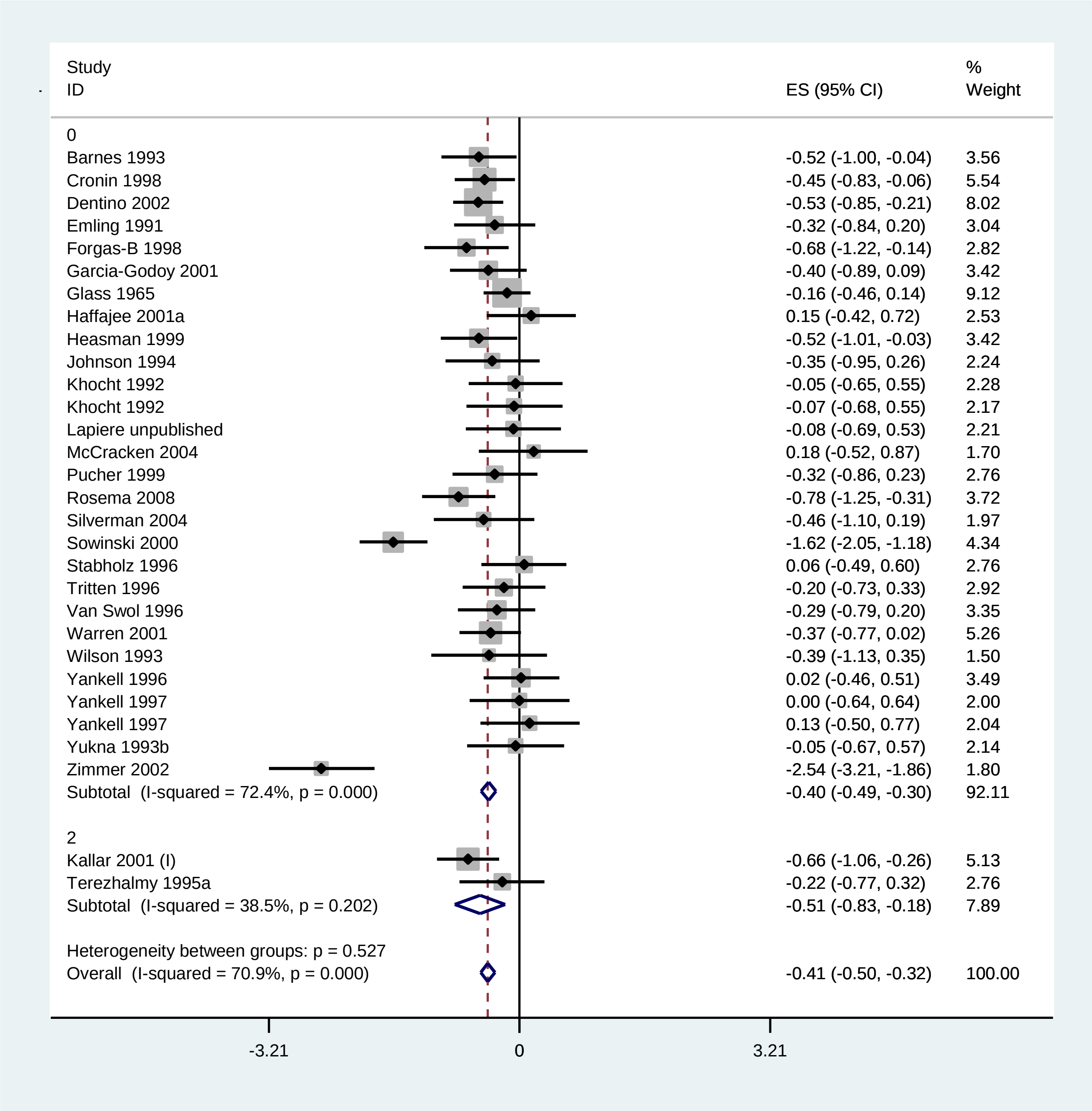

Study
ID
ES (95% CI)
%
Weight
0
Barnes 1993 -0.52 (-1.00, -0.04) 3.56
Cronin 1998 -0.45 (-0.83, -0.06) 5.54
Dentino 2002 -0.53 (-0.85, -0.21) 8.02
Emling 1991 -0.32 (-0.84, 0.20) 3.04
Forgas-B 1998 -0.68 (-1.22, -0.14) 2.82
Garcia-Godoy 2001 -0.40 (-0.89, 0.09) 3.42
Glass 1965 -0.16 (-0.46, 0.14) 9.12
Haffajee 2001a 0.15 (-0.42, 0.72) 2.53
Heasman 1999 -0.52 (-1.01, -0.03) 3.42
Johnson 1994 -0.35 (-0.95, 0.26) 2.24
Khocht 1992 -0.05 (-0.65, 0.55) 2.28
Khocht 1992 -0.07 (-0.68, 0.55) 2.17
Lapiere unpublished -0.08 (-0.69, 0.53) 2.21
McCracken 2004 0.18 (-0.52, 0.87) 1.70
Pucher 1999 -0.32 (-0.86, 0.23) 2.76
Rosema 2008 -0.78 (-1.25, -0.31) 3.72
Silverman 2004 -0.46 (-1.10, 0.19) 1.97
Sowinski 2000 -1.62 (-2.05, -1.18) 4.34
Stabholz 1996 0.06 (-0.49, 0.60) 2.76
Tritten 1996 -0.20 (-0.73, 0.33) 2.92
Van Swol 1996 -0.29 (-0.79, 0.20) 3.35
Warren 2001 -0.37 (-0.77, 0.02) 5.26
Wilson 1993 -0.39 (-1.13, 0.35) 1.50
Yankell 1996 0.02 (-0.46, 0.51) 3.49
Yankell 1997 0.00 (-0.64, 0.64) 2.00
Yankell 1997 0.13 (-0.50, 0.77) 2.04
Yukna 1993b -0.05 (-0.67, 0.57) 2.14
Zimmer 2002 -2.54 (-3.21, -1.86) 1.80
Subtotal (I-squared = 72.4%, p = 0.000) -0.40 (-0.49, -0.30) 92.11
2
Kallar 2001 (I) -0.66 (-1.06, -0.26) 5.13
Terezhalmy 1995a -0.22 (-0.77, 0.32) 2.76
Subtotal (I-squared = 38.5%, p = 0.202) -0.51 (-0.83, -0.18) 7.89
Heterogeneity between groups: p = 0.527
Overall (I-squared = 70.9%, p = 0.000) -0.41 (-0.50, -0.32) 100.00
-3.21
0
3.21

A-5 (4, 7)

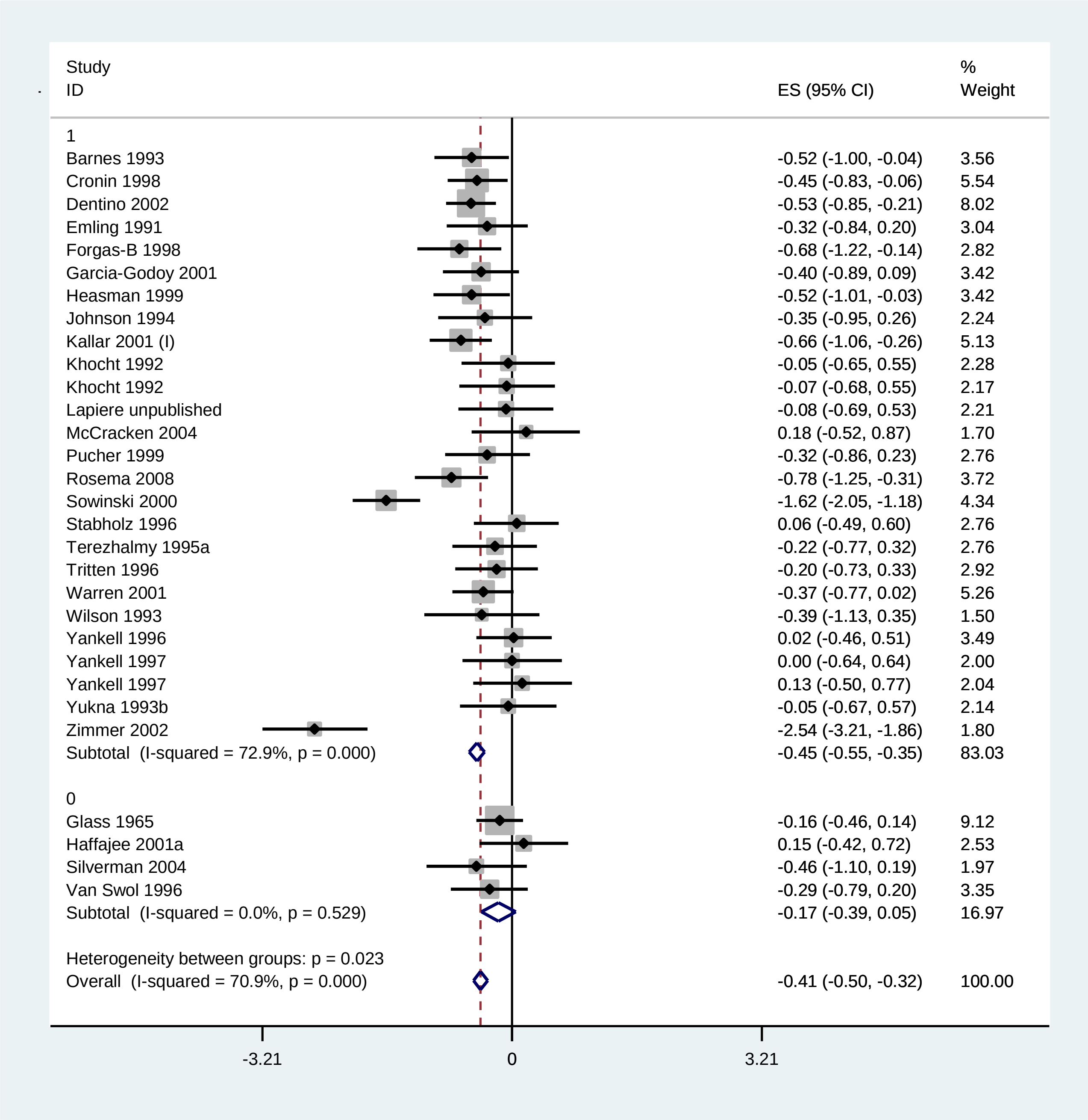

Study ID
ES (95% CI)
% Weight
1
Barnes 1993 -0.52 (-1.00, -0.04) 3.56
Cronin 1998 -0.45 (-0.83, -0.06) 5.54
Dentino 2002 -0.53 (-0.85, -0.21) 8.02
Emling 1991 -0.32 (-0.84, 0.20) 3.04
Forgas-B 1998 -0.68 (-1.22, -0.14) 2.82
Garcia-Godoy 2001 -0.40 (-0.89, 0.09) 3.42
Heasman 1999 -0.52 (-1.01, -0.03) 3.42
Johnson 1994 -0.35 (-0.95, 0.26) 2.24
Kallar 2001 (I) -0.66 (-1.06, -0.26) 5.13
Khocht 1992 -0.05 (-0.65, 0.55) 2.28
Khocht 1992 -0.07 (-0.68, 0.55) 2.17
Lapiere unpublished -0.08 (-0.69, 0.53) 2.21
McCracken 2004 0.18 (-0.52, 0.87) 1.70
Pucher 1999 -0.32 (-0.86, 0.23) 2.76
Rosema 2008 -0.78 (-1.25, -0.31) 3.72
Sowinski 2000 -1.62 (-2.05, -1.18) 4.34
Stabholz 1996 0.06 (-0.49, 0.60) 2.76
Terezhalmy 1995a -0.22 (-0.77, 0.32) 2.76
Tritten 1996 -0.20 (-0.73, 0.33) 2.92
Warren 2001 -0.37 (-0.77, 0.02) 5.26
Wilson 1993 -0.39 (-1.13, 0.35) 1.50
Yankell 1996 0.02 (-0.46, 0.51) 3.49
Yankell 1997 0.00 (-0.64, 0.64) 2.00
Yankell 1997 0.13 (-0.50, 0.77) 2.04
Yukna 1993b -0.05 (-0.67, 0.57) 2.14
Zimmer 2002 -2.54 (-3.21, -1.86) 1.80
Subtotal (I-squared = 72.9%, p = 0.000) -0.45 (-0.55, -0.35) 83.03
0
Glass 1965 -0.16 (-0.46, 0.14) 9.12
Haffajee 2001a 0.15 (-0.42, 0.72) 2.53
Silverman 2004 -0.46 (-1.10, 0.19) 1.97
Van Swol 1996 -0.29 (-0.79, 0.20) 3.35
Subtotal (I-squared = 0.0%, p = 0.529) -0.17 (-0.39, 0.05) 16.97
Heterogeneity between groups: p = 0.023
Overall (I-squared = 70.9%, p = 0.000) -0.41 (-0.50, -0.32) 100.00
-3.21
0
3.21

A-6 (1, 5)

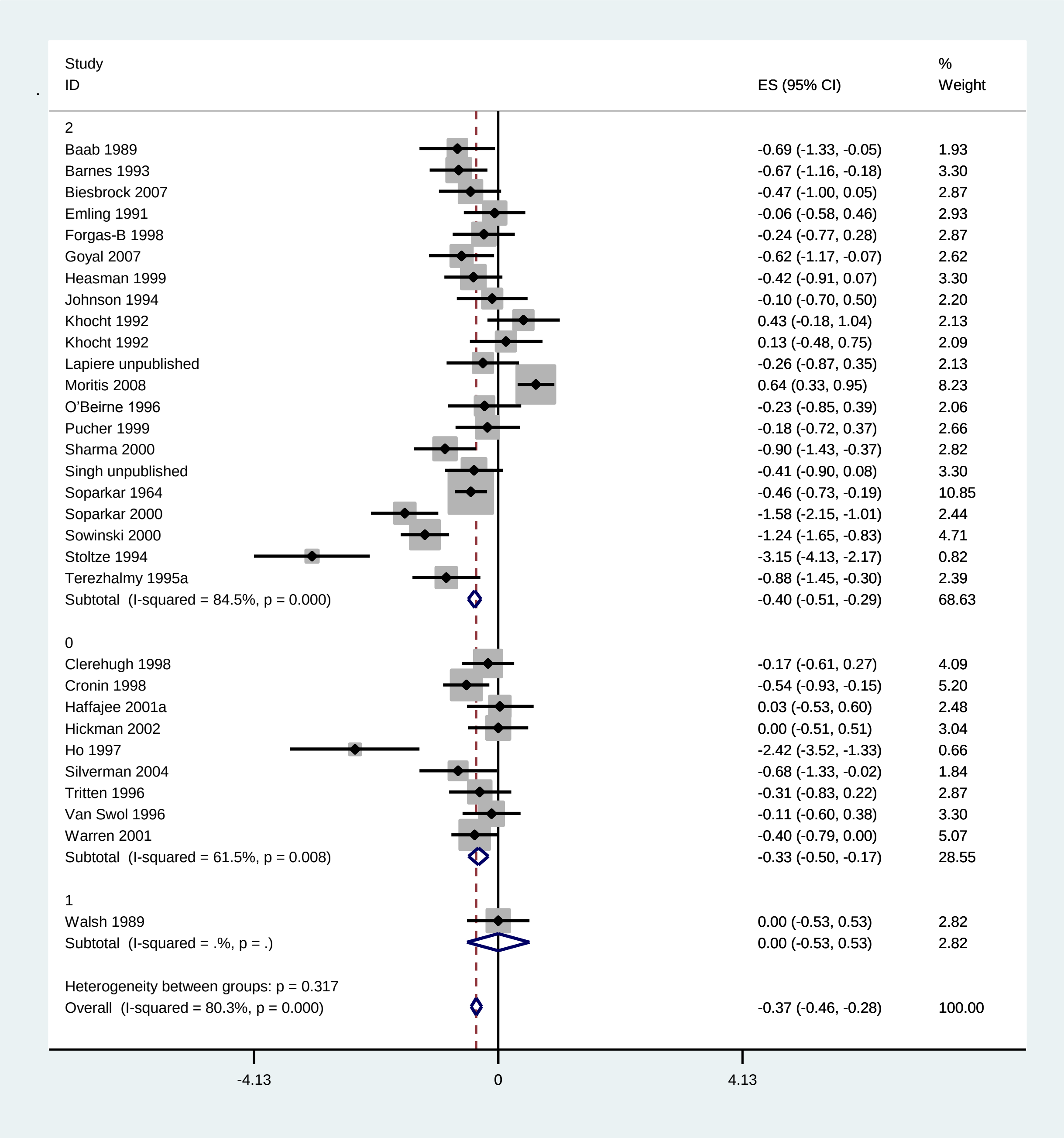

Study
ID
ES (95% CI)
%
Weight
2
Baab 1989 -0.69 (-1.33, -0.05) 1.93
Barnes 1993 -0.67 (-1.16, -0.18) 3.30
Biesbrock 2007 -0.47 (-1.00, 0.05) 2.87
Emling 1991 -0.06 (-0.58, 0.46) 2.93
Forgas-B 1998 -0.24 (-0.77, 0.28) 2.87
Goyal 2007 -0.62 (-1.17, -0.07) 2.62
Heasman 1999 -0.42 (-0.91, 0.07) 3.30
Johnson 1994 -0.10 (-0.70, 0.50) 2.20
Khocht 1992 0.43 (-0.18, 1.04) 2.13
Khocht 1992 0.13 (-0.48, 0.75) 2.09
Lapiere unpublished -0.26 (-0.87, 0.35) 2.13
Moritis 2008 0.64 (0.33, 0.95) 8.23
O'Beirne 1996 -0.23 (-0.85, 0.39) 2.06
Pucher 1999 -0.18 (-0.72, 0.37) 2.66
Sharma 2000 -0.90 (-1.43, -0.37) 2.82
Singh unpublished -0.41 (-0.90, 0.08) 3.30
Soparkar 1964 -0.46 (-0.73, -0.19) 10.85
Soparkar 2000 -1.58 (-2.15, -1.01) 2.44
Sowinski 2000 -1.24 (-1.65, -0.83) 4.71
Stoltze 1994 -3.15 (-4.13, -2.17) 0.82
Terezhalmy 1995a -0.88 (-1.45, -0.30) 2.39
Subtotal (I-squared = 84.5%, p = 0.000) -0.40 (-0.51, -0.29) 68.63
0
Clerehugh 1998 -0.17 (-0.61, 0.27) 4.09
Cronin 1998 -0.54 (-0.93, -0.15) 5.20
Haffajee 2001a 0.03 (-0.53, 0.60) 2.48
Hickman 2002 0.00 (-0.51, 0.51) 3.04
Ho 1997 -2.42 (-3.52, -1.33) 0.66
Silverman 2004 -0.68 (-1.33, -0.02) 1.84
Tritten 1996 -0.31 (-0.83, 0.22) 2.87
Van Swol 1996 -0.11 (-0.60, 0.38) 3.30
Warren 2001 -0.40 (-0.79, 0.00) 5.07
Subtotal (I-squared = 61.5%, p = 0.008) -0.33 (-0.50, -0.17) 28.55
1
Walsh 1989 0.00 (-0.53, 0.53) 2.82
Subtotal (I-squared = .%, p = .) 0.00 (-0.53, 0.53) 2.82
Heterogeneity between groups: p = 0.317
Overall (I-squared = 80.3%, p = 0.000) -0.37 (-0.46, -0.28) 100.00
-4.13
0
4.13

A-6 (2, 6)

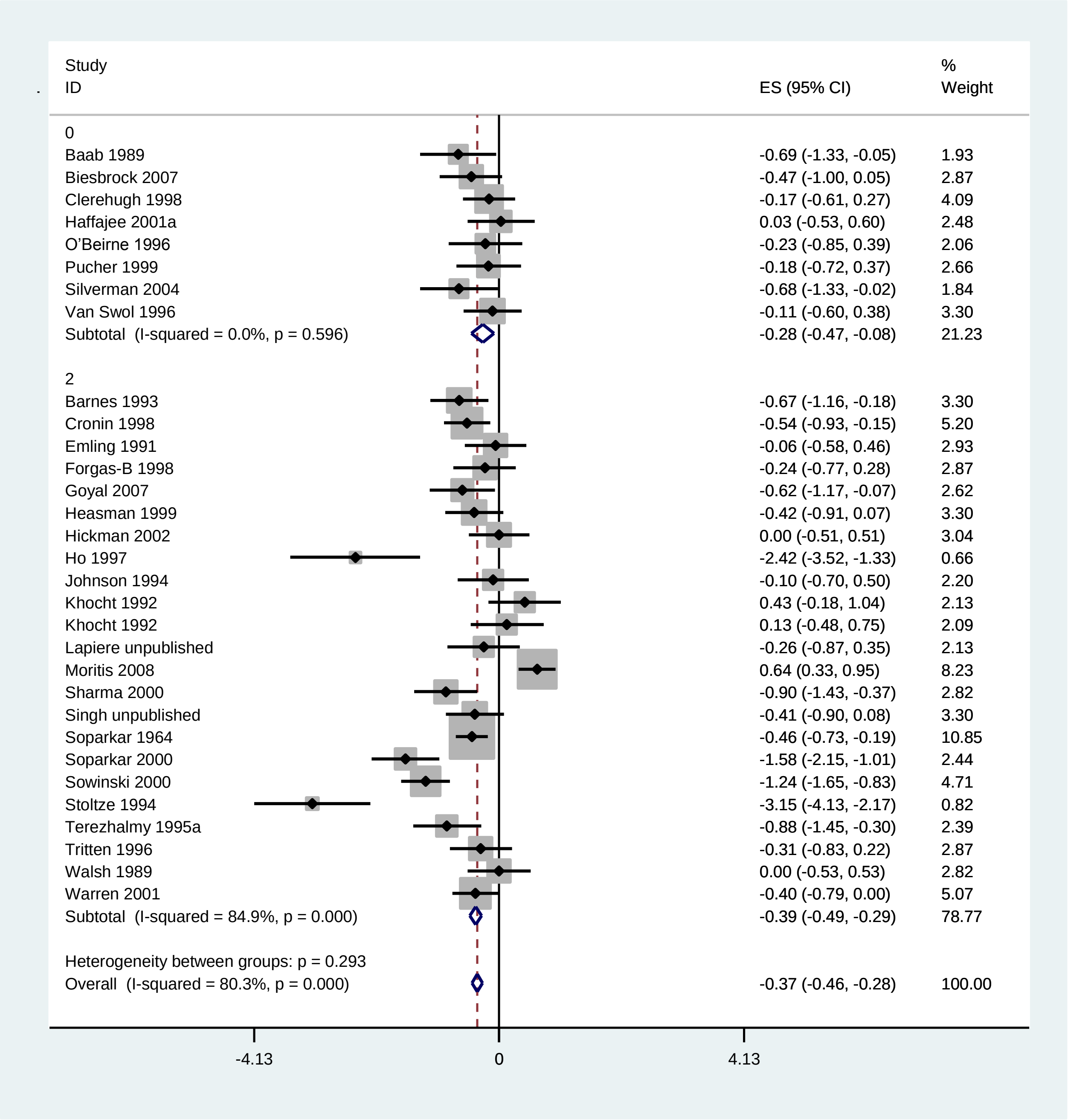

Study
ID
ES (95% CI)
%
Weight
0
Baab 1989 -0.69 (-1.33, -0.05) 1.93
Biesbrock 2007 -0.47 (-1.00, 0.05) 2.87
Clerehugh 1998 -0.17 (-0.61, 0.27) 4.09
Haffajee 2001a 0.03 (-0.53, 0.60) 2.48
O’Beirne 1996 -0.23 (-0.85, 0.39) 2.06
Pucher 1999 -0.18 (-0.72, 0.37) 2.66
Silverman 2004 -0.68 (-1.33, -0.02) 1.84
Van Swol 1996 -0.11 (-0.60, 0.38) 3.30
Subtotal (I-squared = 0.0%, p = 0.596) -0.28 (-0.47, -0.08) 21.23
2
Barnes 1993 -0.67 (-1.16, -0.18) 3.30
Cronin 1998 -0.54 (-0.93, -0.15) 5.20
Emling 1991 -0.06 (-0.58, 0.46) 2.93
Forgas-B 1998 -0.24 (-0.77, 0.28) 2.87
Goyal 2007 -0.62 (-1.17, -0.07) 2.62
Heasman 1999 -0.42 (-0.91, 0.07) 3.30
Hickman 2002 0.00 (-0.51, 0.51) 3.04
Ho 1997 -2.42 (-3.52, -1.33) 0.66
Johnson 1994 -0.10 (-0.70, 0.50) 2.20
Khocht 1992 0.43 (-0.18, 1.04) 2.13
Khocht 1992 0.13 (-0.48, 0.75) 2.09
Lapiere unpublished -0.26 (-0.87, 0.35) 2.13
Moritis 2008 0.64 (0.33, 0.95) 8.23
Sharma 2000 -0.90 (-1.43, -0.37) 2.82
Singh unpublished -0.41 (-0.90, 0.08) 3.30
Soparkar 1964 -0.46 (-0.73, -0.19) 10.85
Soparkar 2000 -1.58 (-2.15, -1.01) 2.44
Sowinski 2000 -1.24 (-1.65, -0.83) 4.71
Stoltze 1994 -3.15 (-4.13, -2.17) 0.82
Terezhalmy 1995a -0.88 (-1.45, -0.30) 2.39
Tritten 1996 -0.31 (-0.83, 0.22) 2.87
Walsh 1989 0.00 (-0.53, 0.53) 2.82
Warren 2001 -0.40 (-0.79, 0.00) 5.07
Subtotal (I-squared = 84.9%, p = 0.000) -0.39 (-0.49, -0.29) 78.77
Heterogeneity between groups: p = 0.293
Overall (I-squared = 80.3%, p = 0.000) -0.37 (-0.46, -0.28) 100.00
-4.13
0
4.13

A-6 (3)

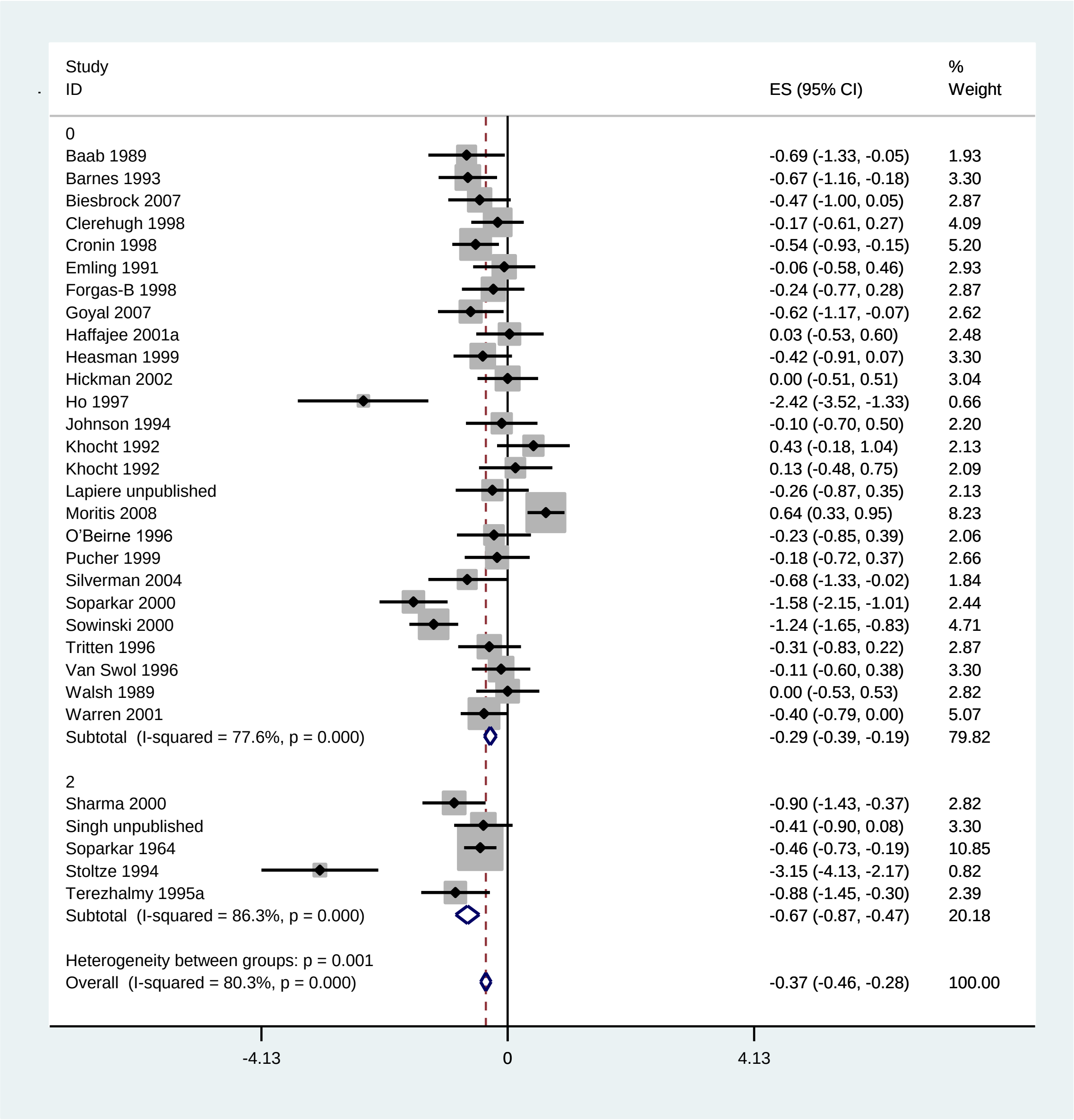
Study
ID
ES (95% CI)
%
Weight
0
Baab 1989 -0.69 (-1.33, -0.05) 1.93
Barnes 1993 -0.67 (-1.16, -0.18) 3.30
Biesbrock 2007 -0.47 (-1.00, 0.05) 2.87
Clerehugh 1998 -0.17 (-0.61, 0.27) 4.09
Cronin 1998 -0.54 (-0.93, -0.15) 5.20
Emling 1991 -0.06 (-0.58, 0.46) 2.93
Forgas-B 1998 -0.24 (-0.77, 0.28) 2.87
Goyal 2007 -0.62 (-1.17, -0.07) 2.62
Haffajee 2001a 0.03 (-0.53, 0.60) 2.48
Heasman 1999 -0.42 (-0.91, 0.07) 3.30
Hickman 2002 0.00 (-0.51, 0.51) 3.04
Ho 1997 -2.42 (-3.52, -1.33) 0.66
Johnson 1994 -0.10 (-0.70, 0.50) 2.20
Khocht 1992 0.43 (-0.18, 1.04) 2.13
Khocht 1992 0.13 (-0.48, 0.75) 2.09
Lapiere unpublished -0.26 (-0.87, 0.35) 2.13
Moritis 2008 0.64 (0.33, 0.95) 8.23
O'Beirne 1996 -0.23 (-0.85, 0.39) 2.06
Pucher 1999 -0.18 (-0.72, 0.37) 2.66
Silverman 2004 -0.68 (-1.33, -0.02) 1.84
Soparkar 2000 -1.58 (-2.15, -1.01) 2.44
Sowinski 2000 -1.24 (-1.65, -0.83) 4.71
Tritten 1996 -0.31 (-0.83, 0.22) 2.87
Van Swol 1996 -0.11 (-0.60, 0.38) 3.30
Walsh 1989 0.00 (-0.53, 0.53) 2.82
Warren 2001 -0.40 (-0.79, 0.00) 5.07
Subtotal (I-squared = 77.6%, p = 0.000) -0.29 (-0.39, -0.19) 79.82
2
Sharma 2000 -0.90 (-1.43, -0.37) 2.82
Singh unpublished -0.41 (-0.90, 0.08) 3.30
Soparkar 1964 -0.46 (-0.73, -0.19) 10.85
Stoltze 1994 -3.15 (-4.13, -2.17) 0.82
Terezhalmy 1995a -0.88 (-1.45, -0.30) 2.39
Subtotal (I-squared = 86.3%, p = 0.000) -0.67 (-0.87, -0.47) 20.18
Heterogeneity between groups: p = 0.001
Overall (I-squared = 80.3%, p = 0.000) -0.37 (-0.46, -0.28) 100.00
-4.13
0
4.13

A-6 (4, 7)

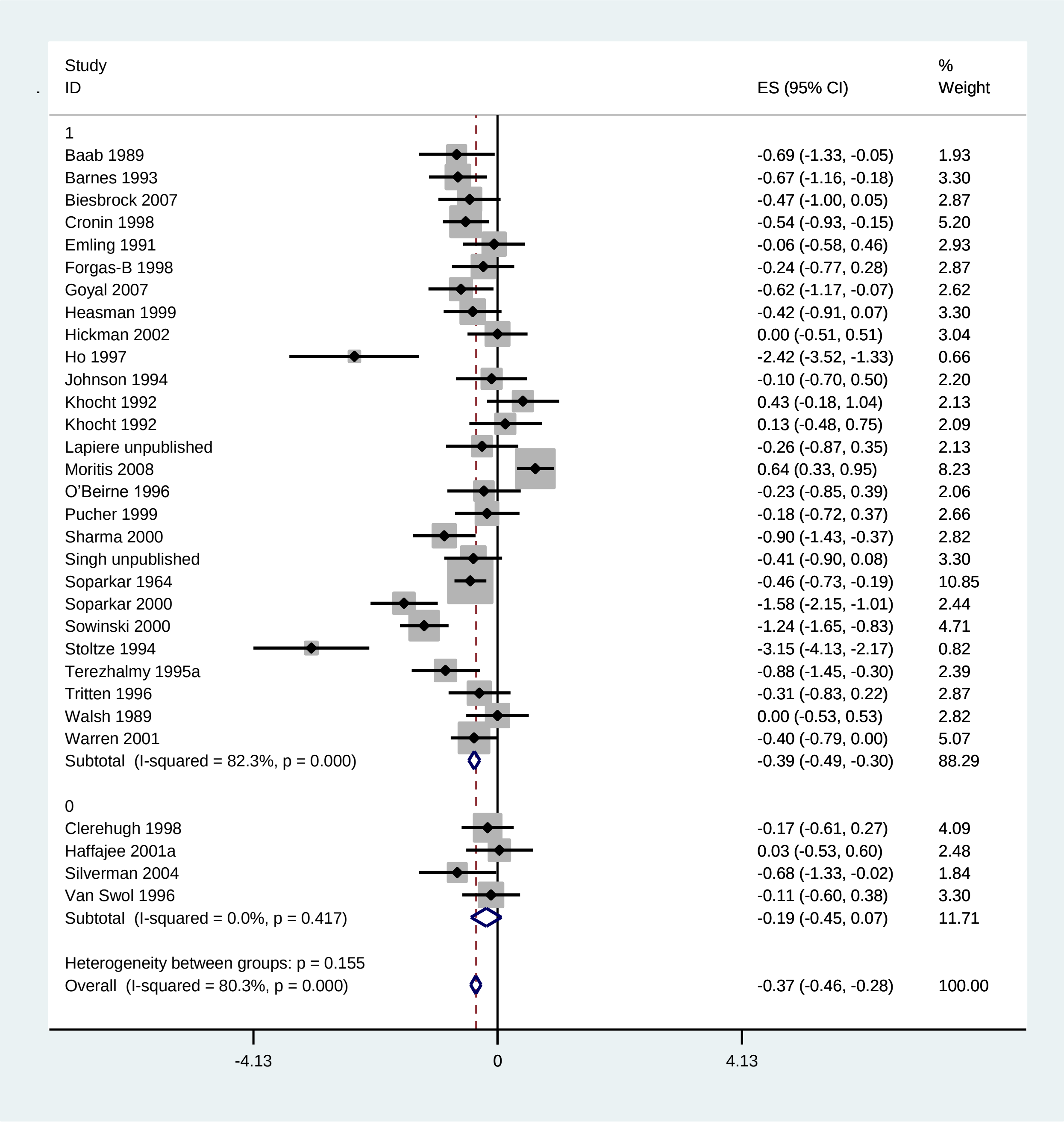

Study
ID
ES (95% CI)
%
Weight
1
Baab 1989 -0.69 (-1.33, -0.05) 1.93
Barnes 1993 -0.67 (-1.16, -0.18) 3.30
Biesbrock 2007 -0.47 (-1.00, 0.05) 2.87
Cronin 1998 -0.54 (-0.93, -0.15) 5.20
Emling 1991 -0.06 (-0.58, 0.46) 2.93
Forgas-B 1998 -0.24 (-0.77, 0.28) 2.87
Goyal 2007 -0.62 (-1.17, -0.07) 2.62
Heasman 1999 -0.42 (-0.91, 0.07) 3.30
Hickman 2002 0.00 (-0.51, 0.51) 3.04
Ho 1997 -2.42 (-3.52, -1.33) 0.66
Johnson 1994 -0.10 (-0.70, 0.50) 2.20
Khocht 1992 0.43 (-0.18, 1.04) 2.13
Khocht 1992 0.13 (-0.48, 0.75) 2.09
Lapiere unpublished -0.26 (-0.87, 0.35) 2.13
Moritis 2008 0.64 (0.33, 0.95) 8.23
O'Beirne 1996 -0.23 (-0.85, 0.39) 2.06
Pucher 1999 -0.18 (-0.72, 0.37) 2.66
Sharma 2000 -0.90 (-1.43, -0.37) 2.82
Singh unpublished -0.41 (-0.90, 0.08) 3.30
Soparkar 1964 -0.46 (-0.73, -0.19) 10.85
Soparkar 2000 -1.58 (-2.15, -1.01) 2.44
Sowinski 2000 -1.24 (-1.65, -0.83) 4.71
Stoltze 1994 -3.15 (-4.13, -2.17) 0.82
Terezhalmy 1995a -0.88 (-1.45, -0.30) 2.39
Tritten 1996 -0.31 (-0.83, 0.22) 2.87
Walsh 1989 0.00 (-0.53, 0.53) 2.82
Warren 2001 -0.40 (-0.79, 0.00) 5.07
Subtotal (I-squared = 82.3%, p = 0.000) -0.39 (-0.49, -0.30) 88.29
0
Clerehugh 1998 -0.17 (-0.61, 0.27) 4.09
Haffajee 2001a 0.03 (-0.53, 0.60) 2.48
Silverman 2004 -0.68 (-1.33, -0.02) 1.84
Van Swol 1996 -0.11 (-0.60, 0.38) 3.30
Subtotal (I-squared = 0.0%, p = 0.417) -0.19 (-0.45, 0.07) 11.71
Heterogeneity between groups: p = 0.155
Overall (I-squared = 80.3%, p = 0.000) -0.37 (-0.46, -0.28) 100.00
-4.13
0
4.13

A-7 (1, 5)

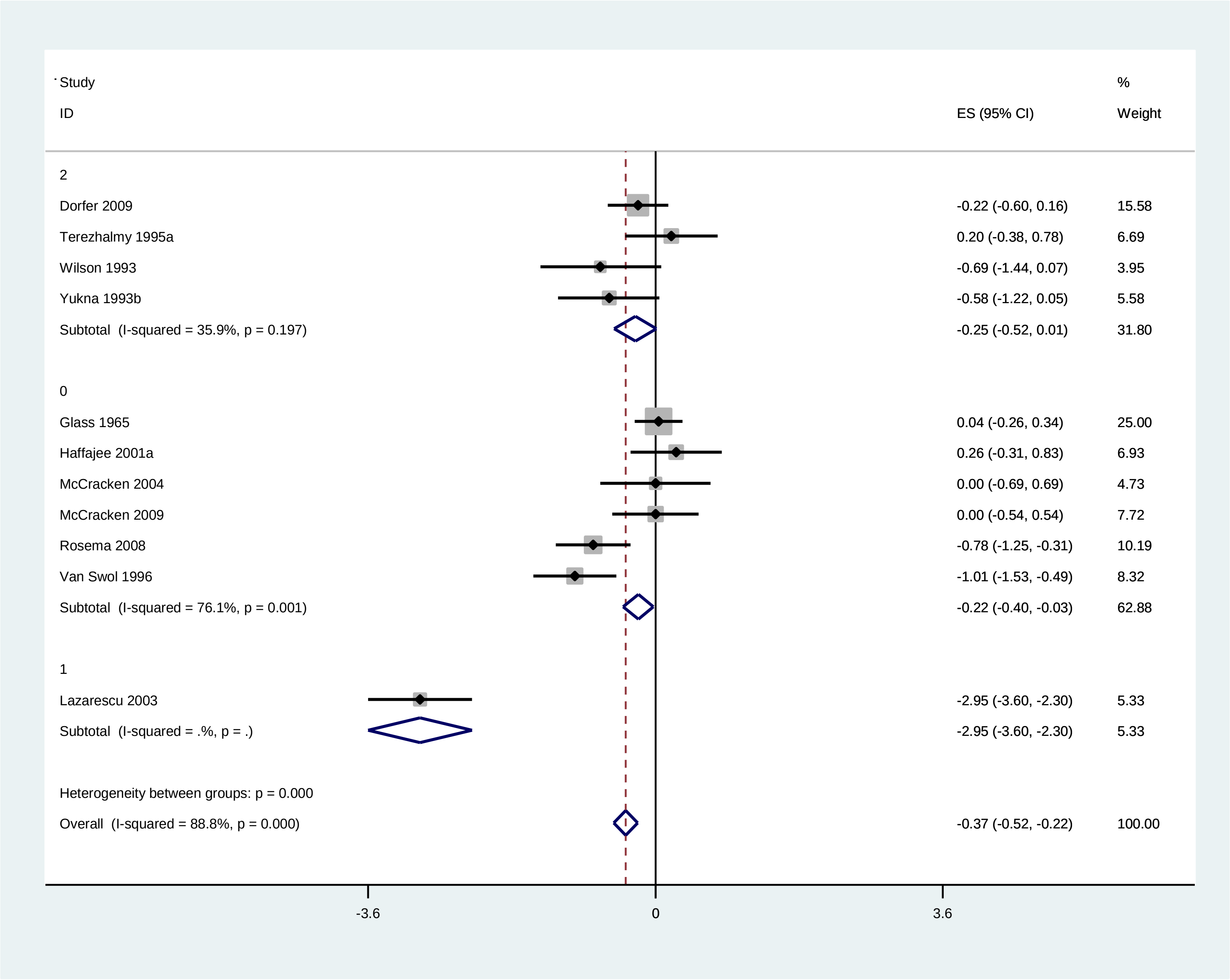
Study
ID
ES (95% CI)
%
Weight
2
Dorfer 2009
-0.22 (-0.60, 0.16)
15.58
Terezhalmy 1995a
0.20 (-0.38, 0.78)
6.69
Wilson 1993
-0.69 (-1.44, 0.07)
3.95
Yukna 1993b
-0.58 (-1.22, 0.05)
5.58
Subtotal (I-squared = 35.9%, p = 0.197)
-0.25 (-0.52, 0.01)
31.80
0
Glass 1965
0.04 (-0.26, 0.34)
25.00
Haffajee 2001a
0.26 (-0.31, 0.83)
6.93
McCracken 2004
0.00 (-0.69, 0.69)
4.73
McCracken 2009
0.00 (-0.54, 0.54)
7.72
Rosema 2008
-0.78 (-1.25, -0.31)
10.19
Van Swol 1996
-1.01 (-1.53, -0.49)
8.32
Subtotal (I-squared = 76.1%, p = 0.001)
-0.22 (-0.40, -0.03)
62.88
1
Lazarescu 2003
-2.95 (-3.60, -2.30)
5.33
Subtotal (I-squared = .%, p = .)
-2.95 (-3.60, -2.30)
5.33
Heterogeneity between groups: p = 0.000
Overall (I-squared = 88.8%, p = 0.000)
-0.37 (-0.52, -0.22)
100.00
-3.6
0
3.6

A-7 (2, 4, 6, 7)

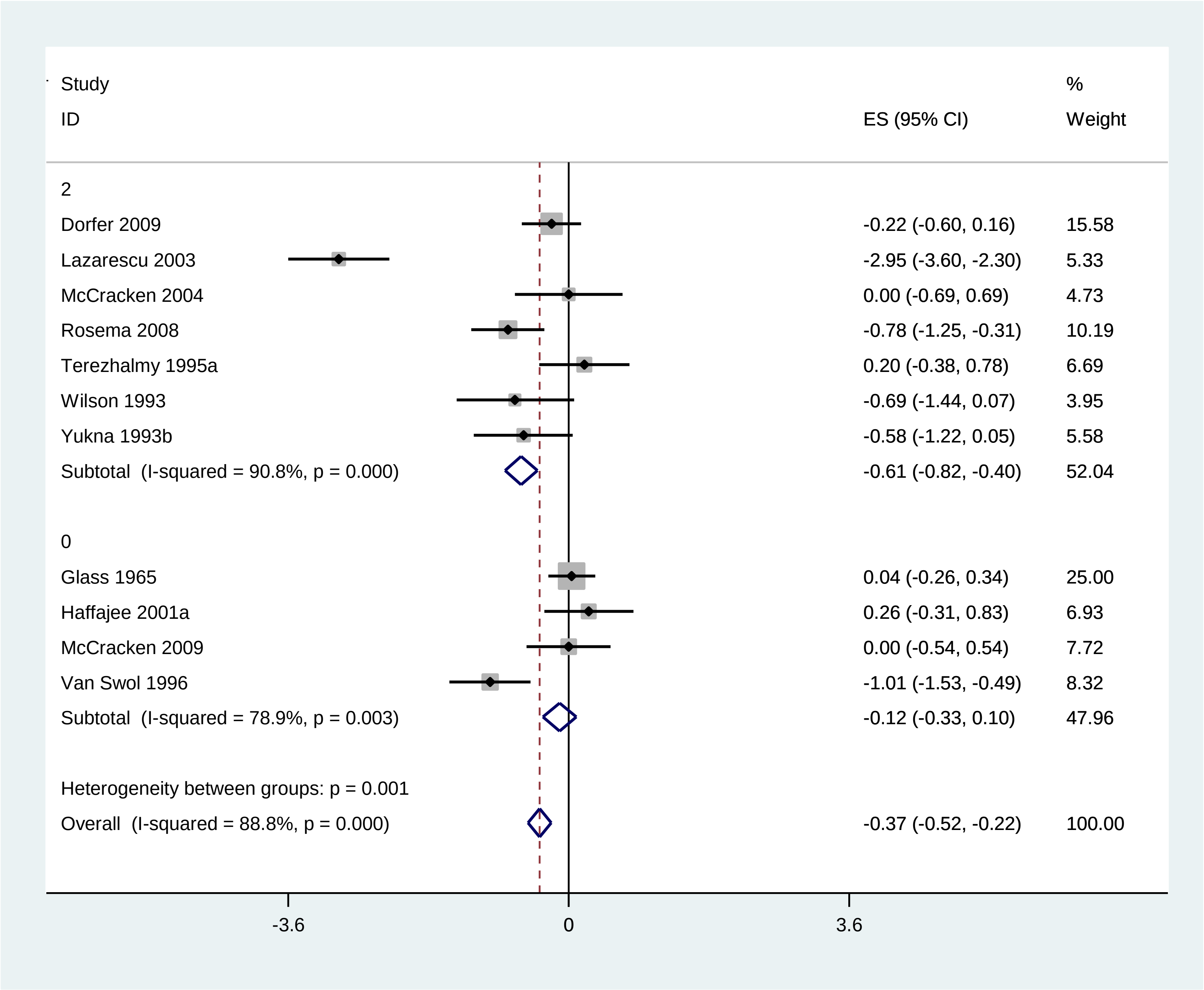
Study
ID
ES (95% CI)
%
Weight
2
Dorfer 2009 -0.22 (-0.60, 0.16) 15.58
Lazarescu 2003 -2.95 (-3.60, -2.30) 5.33
McCracken 2004 0.00 (-0.69, 0.69) 4.73
Rosema 2008 -0.78 (-1.25, -0.31) 10.19
Terezhalmy 1995a 0.20 (-0.38, 0.78) 6.69
Wilson 1993 -0.69 (-1.44, 0.07) 3.95
Yukna 1993b -0.58 (-1.22, 0.05) 5.58
Subtotal (I-squared = 90.8%, p = 0.000) -0.61 (-0.82, -0.40) 52.04
0
Glass 1965 0.04 (-0.26, 0.34) 25.00
Haffajee 2001a 0.26 (-0.31, 0.83) 6.93
McCracken 2009 0.00 (-0.54, 0.54) 7.72
Van Swol 1996 -1.01 (-1.53, -0.49) 8.32
Subtotal (I-squared = 78.9%, p = 0.003) -0.12 (-0.33, 0.10) 47.96
Heterogeneity between groups: p = 0.001
Overall (I-squared = 88.8%, p = 0.000) -0.37 (-0.52, -0.22) 100.00
-3.6
0
3.6

A-7 (3)

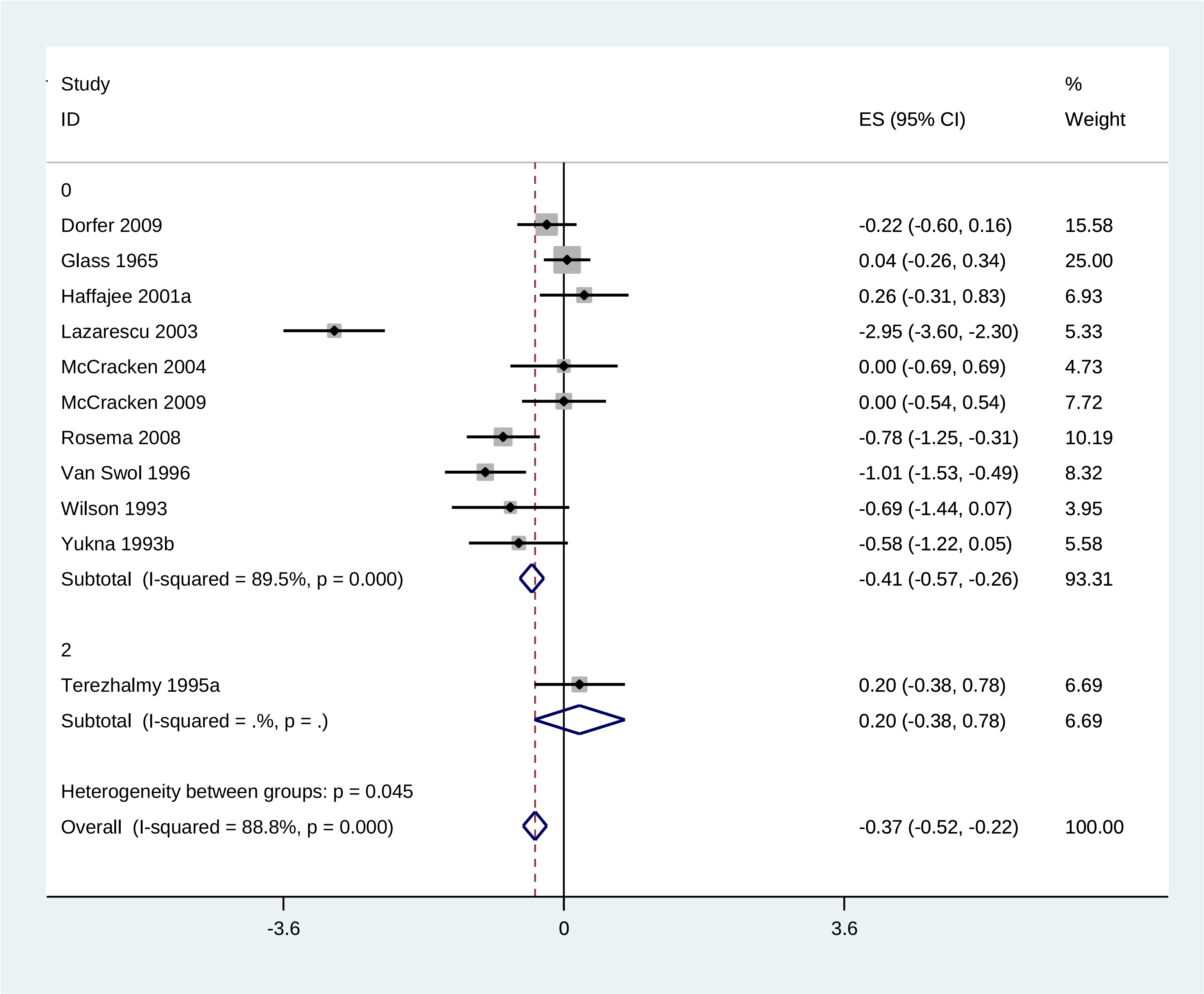

Study
ID
ES (95% CI)
%
Weight
0
Dorfer 2009
-0.22 (-0.60, 0.16)
15.58
Glass 1965
0.04 (-0.26, 0.34)
25.00
Haffajee 2001a
0.26 (-0.31, 0.83)
6.93
Lazarescu 2003
-2.95 (-3.60, -2.30)
5.33
McCracken 2004
0.00 (-0.69, 0.69)
4.73
McCracken 2009
0.00 (-0.54, 0.54)
7.72
Rosema 2008
-0.78 (-1.25, -0.31)
10.19
Van Swol 1996
-1.01 (-1.53, -0.49)
8.32
Wilson 1993
-0.69 (-1.44, 0.07)
3.95
Yukna 1993b
-0.58 (-1.22, 0.05)
5.58
Subtotal (I-squared = 89.5%, p = 0.000)
-0.41 (-0.57, -0.26)
93.31
2
Terezhalmy 1995a
0.20 (-0.38, 0.78)
6.69
Subtotal (I-squared = .%, p = .)
0.20 (-0.38, 0.78)
6.69
Heterogeneity between groups: p = 0.045
Overall (I-squared = 88.8%, p = 0.000)
-0.37 (-0.52, -0.22)
100.00
-3.6
0
3.6

A-8 (1, 5)

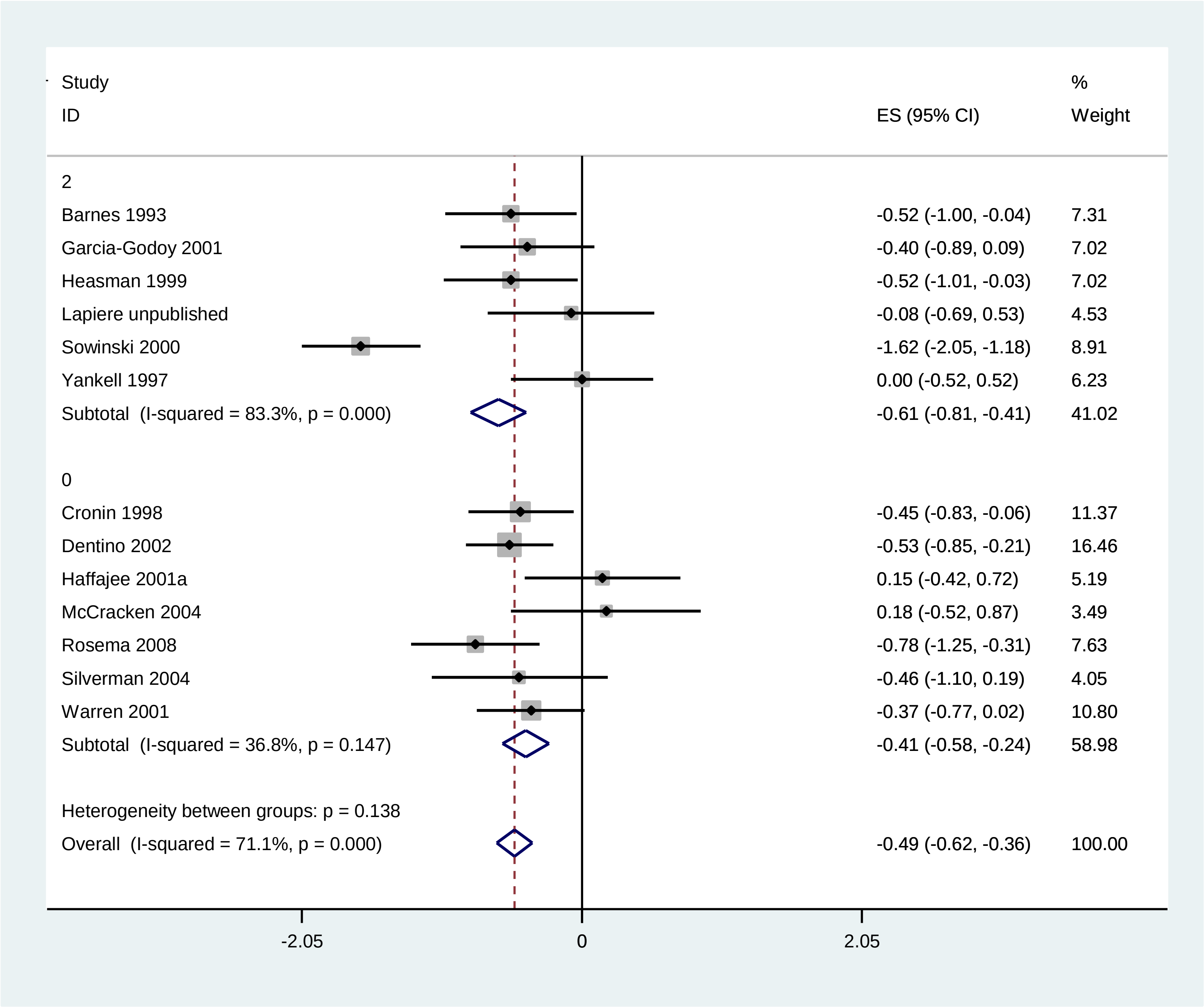
Study
ID
ES (95% CI)
%
Weight
2
Barnes 1993
-0.52 (-1.00, -0.04)
7.31
Garcia-Godoy 2001
-0.40 (-0.89, 0.09)
7.02
Heasman 1999
-0.52 (-1.01, -0.03)
7.02
Lapiere unpublished
-0.08 (-0.69, 0.53)
4.53
Sowinski 2000
-1.62 (-2.05, -1.18)
8.91
Yankell 1997
0.00 (-0.52, 0.52)
6.23
Subtotal (I-squared = 83.3%, p = 0.000)
-0.61 (-0.81, -0.41)
41.02
0
Cronin 1998
-0.45 (-0.83, -0.06)
11.37
Dentino 2002
-0.53 (-0.85, -0.21)
16.46
Haffajee 2001a
0.15 (-0.42, 0.72)
5.19
McCracken 2004
0.18 (-0.52, 0.87)
3.49
Rosema 2008
-0.78 (-1.25, -0.31)
7.63
Silverman 2004
-0.46 (-1.10, 0.19)
4.05
Warren 2001
-0.37 (-0.77, 0.02)
10.80
Subtotal (I-squared = 36.8%, p = 0.147)
-0.41 (-0.58, -0.24)
58.98
Heterogeneity between groups: p = 0.138
Overall (I-squared = 71.1%, p = 0.000)
-0.49 (-0.62, -0.36)
100.00
-2.05
0
2.05

A-8 (2, 4, 6, 7)

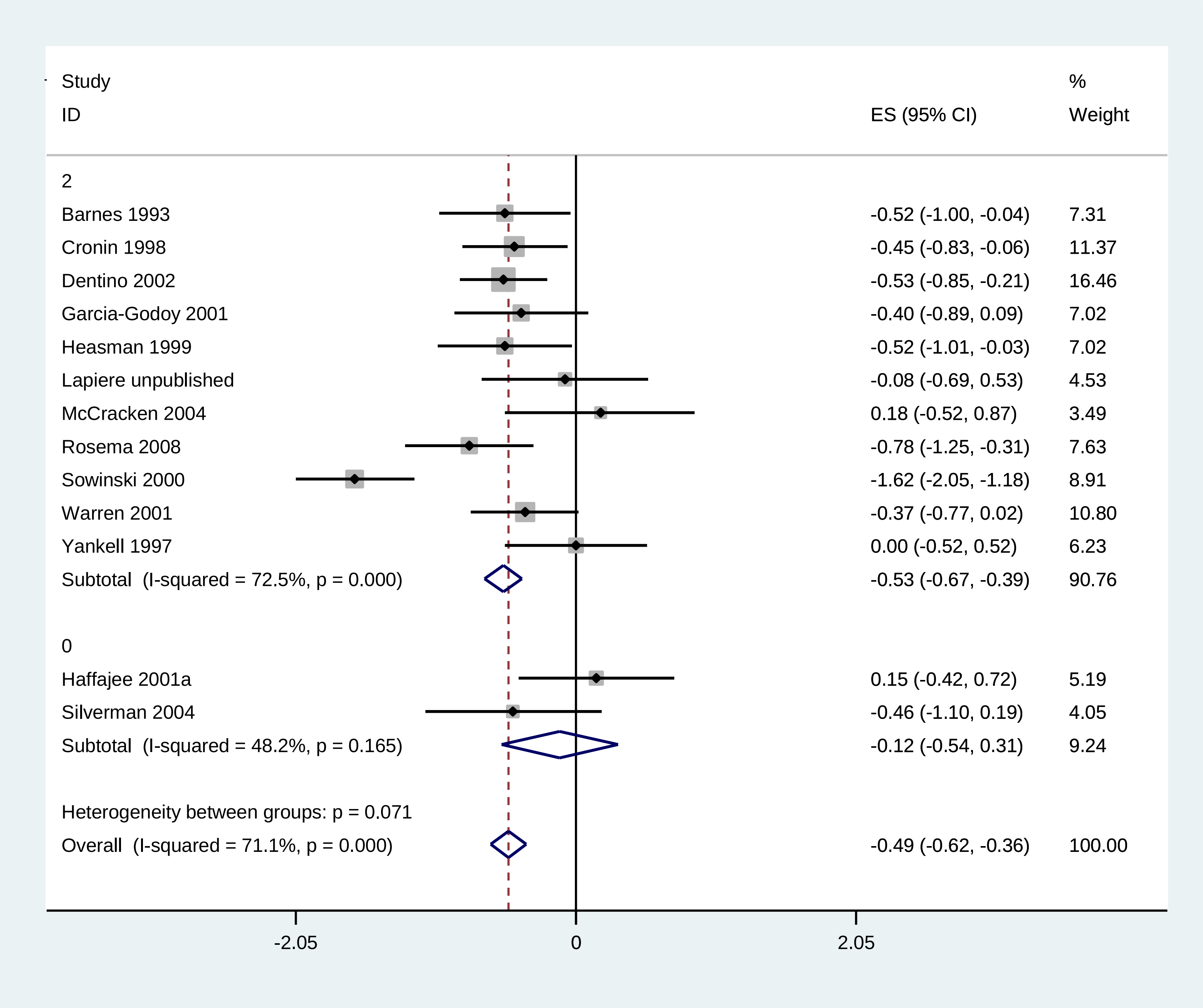
Study
ID
ES (95% CI)
%
Weight
2
Barnes 1993 -0.52 (-1.00, -0.04) 7.31
Cronin 1998 -0.45 (-0.83, -0.06) 11.37
Dentino 2002 -0.53 (-0.85, -0.21) 16.46
Garcia-Godoy 2001 -0.40 (-0.89, 0.09) 7.02
Heasman 1999 -0.52 (-1.01, -0.03) 7.02
Lapiere unpublished -0.08 (-0.69, 0.53) 4.53
McCracken 2004 0.18 (-0.52, 0.87) 3.49
Rosema 2008 -0.78 (-1.25, -0.31) 7.63
Sowinski 2000 -1.62 (-2.05, -1.18) 8.91
Warren 2001 -0.37 (-0.77, 0.02) 10.80
Yankell 1997 0.00 (-0.52, 0.52) 6.23
Subtotal (I-squared = 72.5%, p = 0.000) -0.53 (-0.67, -0.39) 90.76
0
Haffajee 2001a 0.15 (-0.42, 0.72) 5.19
Silverman 2004 -0.46 (-1.10, 0.19) 4.05
Subtotal (I-squared = 48.2%, p = 0.165) -0.12 (-0.54, 0.31) 9.24
Heterogeneity between groups: p = 0.071
Overall (I-squared = 71.1%, p = 0.000) -0.49 (-0.62, -0.36) 100.00
-2.05
0
2.05

A-9 (1, 5)

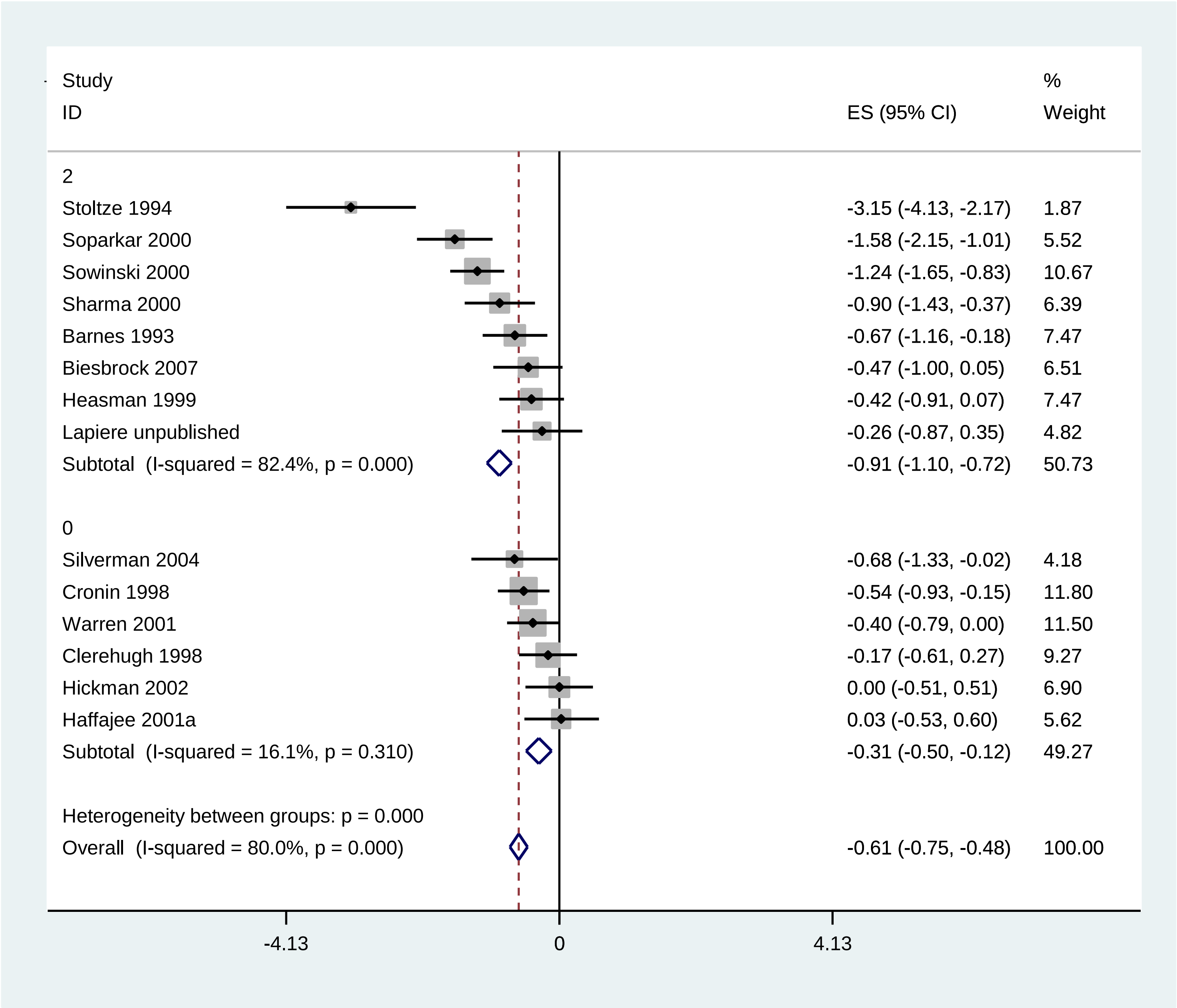
Study
ID
ES (95% CI)
%
Weight
2
Stoltze 1994 -3.15 (-4.13, -2.17) 1.87
Soparkar 2000 -1.58 (-2.15, -1.01) 5.52
Sowinski 2000 -1.24 (-1.65, -0.83) 10.67
Sharma 2000 -0.90 (-1.43, -0.37) 6.39
Barnes 1993 -0.67 (-1.16, -0.18) 7.47
Biesbrock 2007 -0.47 (-1.00, 0.05) 6.51
Heasman 1999 -0.42 (-0.91, 0.07) 7.47
Lapiere unpublished -0.26 (-0.87, 0.35) 4.82
Subtotal (I-squared = 82.4%, p = 0.000) -0.91 (-1.10, -0.72) 50.73
0
Silverman 2004 -0.68 (-1.33, -0.02) 4.18
Cronin 1998 -0.54 (-0.93, -0.15) 11.80
Warren 2001 -0.40 (-0.79, 0.00) 11.50
Clerehugh 1998 -0.17 (-0.61, 0.27) 9.27
Hickman 2002 0.00 (-0.51, 0.51) 6.90
Haffajee 2001a 0.03 (-0.53, 0.60) 5.62
Subtotal (I-squared = 16.1%, p = 0.310) -0.31 (-0.50, -0.12) 49.27
Heterogeneity between groups: p = 0.000
Overall (I-squared = 80.0%, p = 0.000) -0.61 (-0.75, -0.48) 100.00
-4.13
0
4.13

A-9 (2, 6)

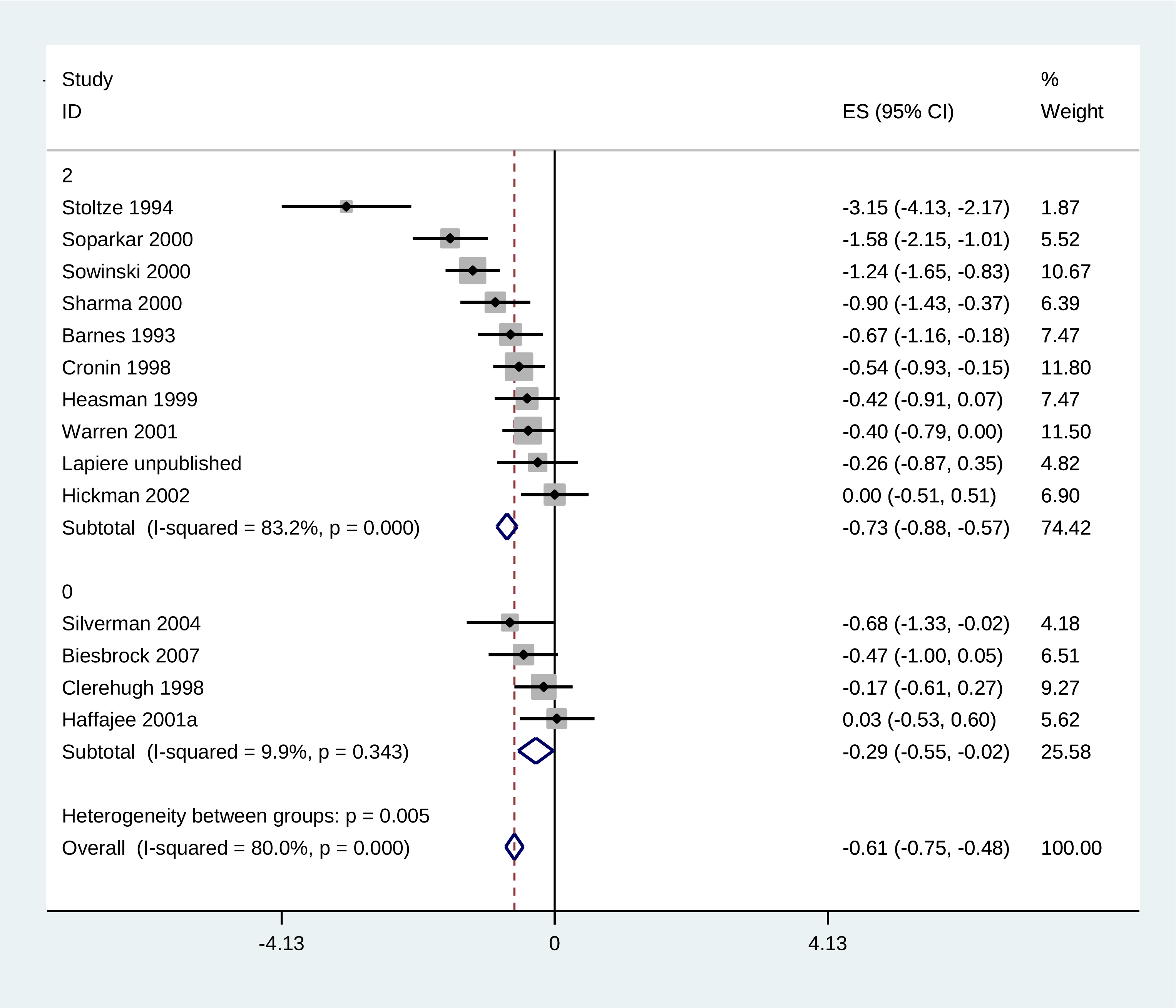

Study
ID
ES (95% CI)
%
Weight
2
Stoltze 1994
-3.15 (-4.13, -2.17)
1.87
Soparkar 2000
-1.58 (-2.15, -1.01)
5.52
Sowinski 2000
-1.24 (-1.65, -0.83)
10.67
Sharma 2000
-0.90 (-1.43, -0.37)
6.39
Barnes 1993
-0.67 (-1.16, -0.18)
7.47
Cronin 1998
-0.54 (-0.93, -0.15)
11.80
Heasman 1999
-0.42 (-0.91, 0.07)
7.47
Warren 2001
-0.40 (-0.79, 0.00)
11.50
Lapiere unpublished
-0.26 (-0.87, 0.35)
4.82
Hickman 2002
0.00 (-0.51, 0.51)
6.90
Subtotal (I-squared = 83.2%, p = 0.000)
-0.73 (-0.88, -0.57)
74.42
0
Silverman 2004
-0.68 (-1.33, -0.02)
4.18
Biesbrock 2007
-0.47 (-1.00, 0.05)
6.51
Clerehugh 1998
-0.17 (-0.61, 0.27)
9.27
Haffajee 2001a
0.03 (-0.53, 0.60)
5.62
Subtotal (I-squared = 9.9%, p = 0.343)
-0.29 (-0.55, -0.02)
25.58
Heterogeneity between groups: p = 0.005
Overall (I-squared = 80.0%, p = 0.000)
-0.61 (-0.75, -0.48)
100.00
-4.13
0
4.13

A-9 (3)

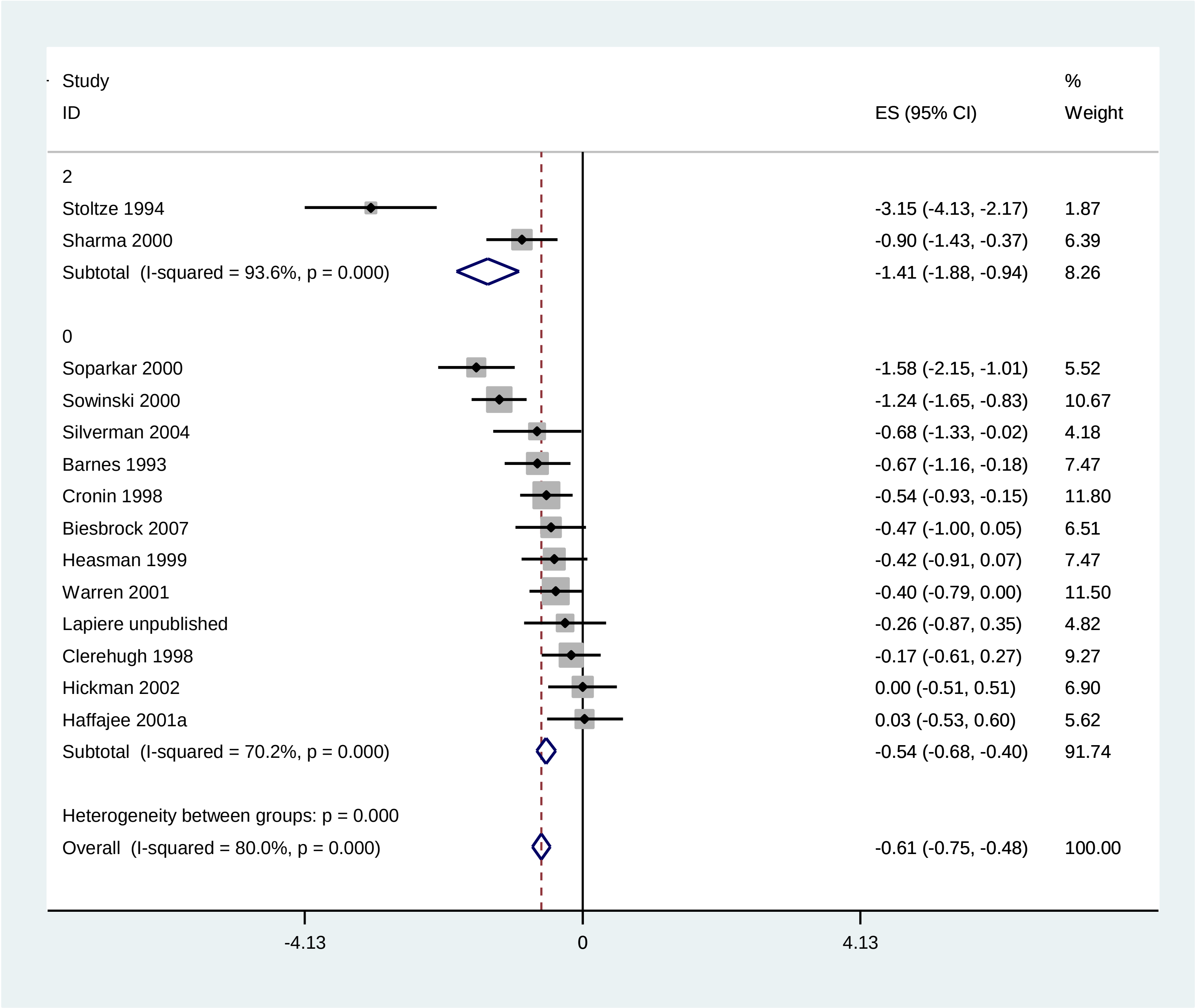
Study
ID
ES (95% CI)
%
Weight
2
Stoltze 1994 -3.15 (-4.13, -2.17) 1.87
Sharma 2000 -0.90 (-1.43, -0.37) 6.39
Subtotal (I-squared = 93.6%, p = 0.000) -1.41 (-1.88, -0.94) 8.26
0
Soparkar 2000 -1.58 (-2.15, -1.01) 5.52
Sowinski 2000 -1.24 (-1.65, -0.83) 10.67
Silverman 2004 -0.68 (-1.33, -0.02) 4.18
Barnes 1993 -0.67 (-1.16, -0.18) 7.47
Cronin 1998 -0.54 (-0.93, -0.15) 11.80
Biesbrock 2007 -0.47 (-1.00, 0.05) 6.51
Heasman 1999 -0.42 (-0.91, 0.07) 7.47
Warren 2001 -0.40 (-0.79, 0.00) 11.50
Lapiere unpublished -0.26 (-0.87, 0.35) 4.82
Clerehugh 1998 -0.17 (-0.61, 0.27) 9.27
Hickman 2002 0.00 (-0.51, 0.51) 6.90
Haffajee 2001a 0.03 (-0.53, 0.60) 5.62
Subtotal (I-squared = 70.2%, p = 0.000) -0.54 (-0.68, -0.40) 91.74
Heterogeneity between groups: p = 0.000
Overall (I-squared = 80.0%, p = 0.000) -0.61 (-0.75, -0.48) 100.00
-4.13
0
4.13

A-9 (4, 7)

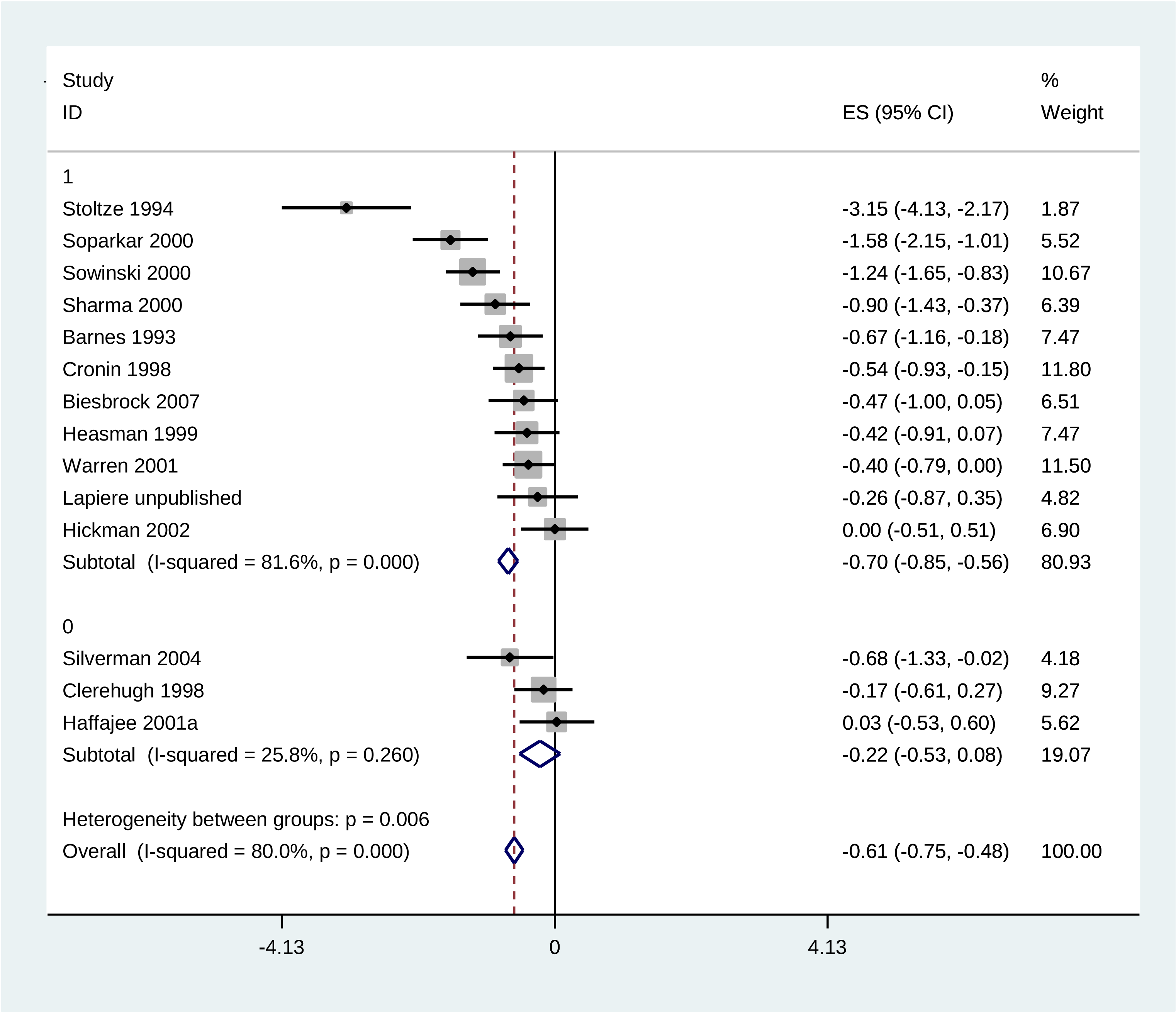
Study
ID
ES (95% CI)
%
Weight
1
Stoltze 1994 -3.15 (-4.13, -2.17) 1.87
Soparkar 2000 -1.58 (-2.15, -1.01) 5.52
Sowinski 2000 -1.24 (-1.65, -0.83) 10.67
Sharma 2000 -0.90 (-1.43, -0.37) 6.39
Barnes 1993 -0.67 (-1.16, -0.18) 7.47
Cronin 1998 -0.54 (-0.93, -0.15) 11.80
Biesbrock 2007 -0.47 (-1.00, 0.05) 6.51
Heasman 1999 -0.42 (-0.91, 0.07) 7.47
Warren 2001 -0.40 (-0.79, 0.00) 11.50
Lapiere unpublished -0.26 (-0.87, 0.35) 4.82
Hickman 2002 0.00 (-0.51, 0.51) 6.90
Subtotal (I-squared = 81.6%, p = 0.000) -0.70 (-0.85, -0.56) 80.93
0
Silverman 2004 -0.68 (-1.33, -0.02) 4.18
Clerehugh 1998 -0.17 (-0.61, 0.27) 9.27
Haffajee 2001a 0.03 (-0.53, 0.60) 5.62
Subtotal (I-squared = 25.8%, p = 0.260) -0.22 (-0.53, 0.08) 19.07
Heterogeneity between groups: p = 0.006
Overall (I-squared = 80.0%, p = 0.000) -0.61 (-0.75, -0.48) 100.00
-4.13
0
4.13

Esposito et al. 2013

B-1 (1)

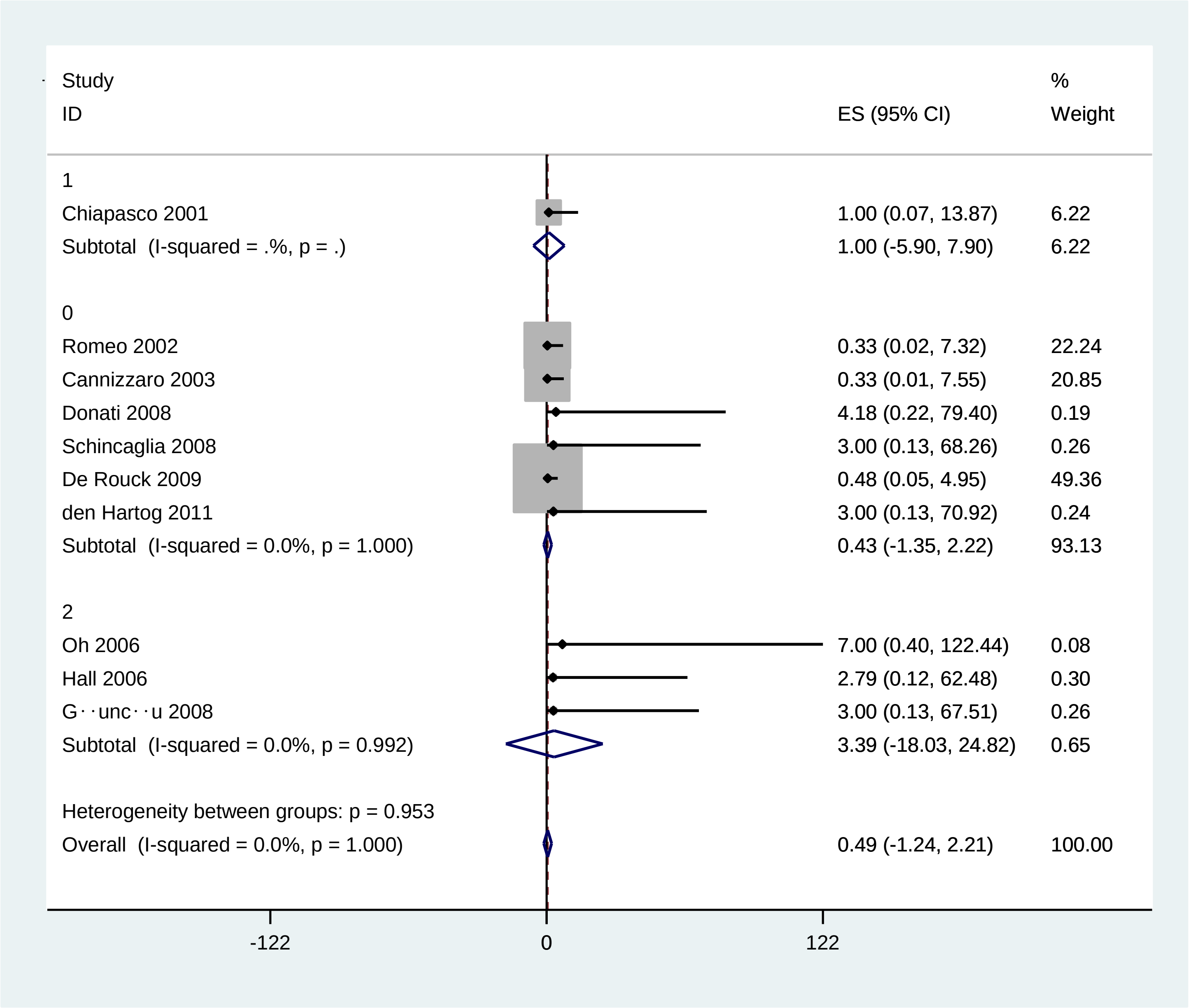

B-1 (2, 4)

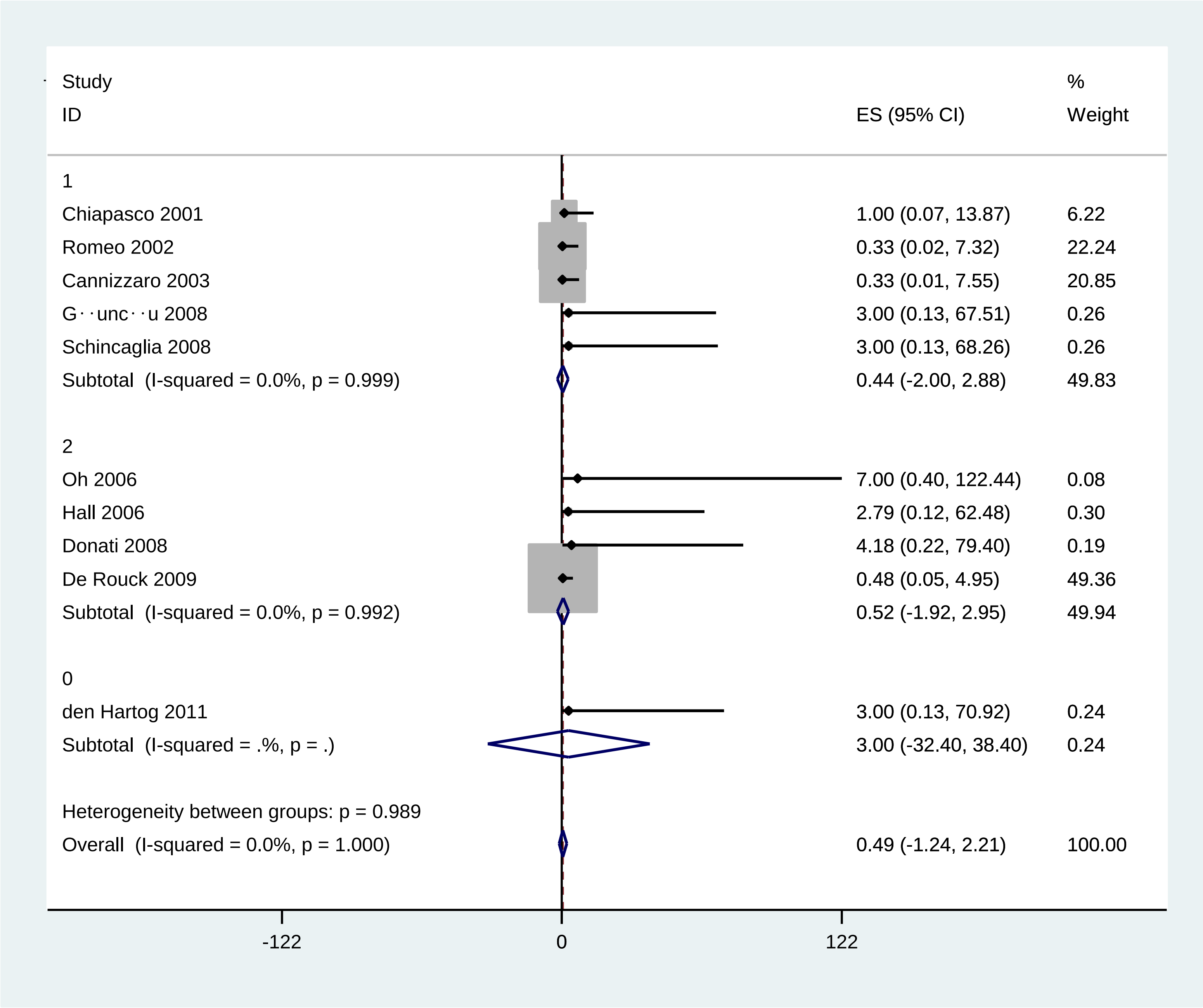

Study
ID
ES (95% CI)
%
Weight
1
Chiapasco 2001
1.00 (0.07, 13.87)
6.22
Romeo 2002
0.33 (0.02, 7.32)
22.24
Cannizzaro 2003
0.33 (0.01, 7.55)
20.85
G· ·unc· ·u 2008
3.00 (0.13, 67.51)
0.26
Schincaglia 2008
3.00 (0.13, 68.26)
0.26
Subtotal (I-squared = 0.0%, p = 0.999)
0.44 (-2.00, 2.88)
49.83
2
Oh 2006
7.00 (0.40, 122.44)
0.08
Hall 2006
2.79 (0.12, 62.48)
0.30
Donati 2008
4.18 (0.22, 79.40)
0.19
De Rouck 2009
0.48 (0.05, 4.95)
49.36
Subtotal (I-squared = 0.0%, p = 0.992)
0.52 (-1.92, 2.95)
49.94
0
den Hartog 2011
3.00 (0.13, 70.92)
0.24
Subtotal (I-squared = .%, p = .)
3.00 (-32.40, 38.40)
0.24
Heterogeneity between groups: p = 0.989
Overall (I-squared = 0.0%, p = 1.000)
0.49 (-1.24, 2.21)
100.00
-122
0
122

B-1 (3)

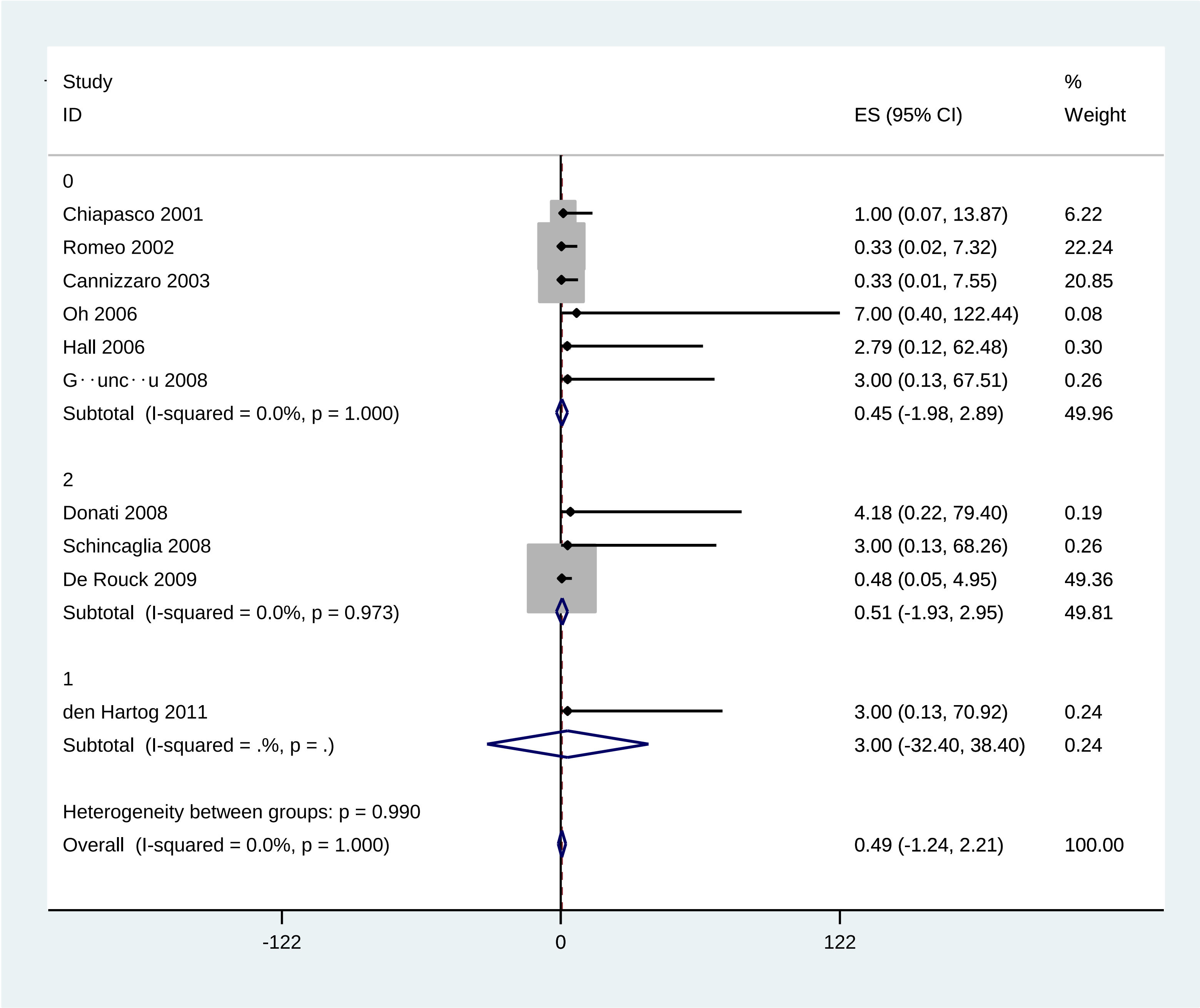

Study
ID
ES (95% CI)
%
Weight
0
Chiapasco 2001 1.00 (0.07, 13.87) 6.22
Romeo 2002 0.33 (0.02, 7.32) 22.24
Cannizzaro 2003 0.33 (0.01, 7.55) 20.85
Oh 2006 7.00 (0.40, 122.44) 0.08
Hall 2006 2.79 (0.12, 62.48) 0.30
G· ·unc· ·u 2008 3.00 (0.13, 67.51) 0.26
Subtotal (I-squared = 0.0%, p = 1.000) 0.45 (-1.98, 2.89) 49.96
2
Donati 2008 4.18 (0.22, 79.40) 0.19
Schincaglia 2008 3.00 (0.13, 68.26) 0.26
De Rouck 2009 0.48 (0.05, 4.95) 49.36
Subtotal (I-squared = 0.0%, p = 0.973) 0.51 (-1.93, 2.95) 49.81
1
den Hartog 2011 3.00 (0.13, 70.92) 0.24
Subtotal (I-squared = .%, p = .) 3.00 (-32.40, 38.40) 0.24
Heterogeneity between groups: p = 0.990
Overall (I-squared = 0.0%, p = 1.000) 0.49 (-1.24, 2.21) 100.00
-122
0
122

B-1 (5)

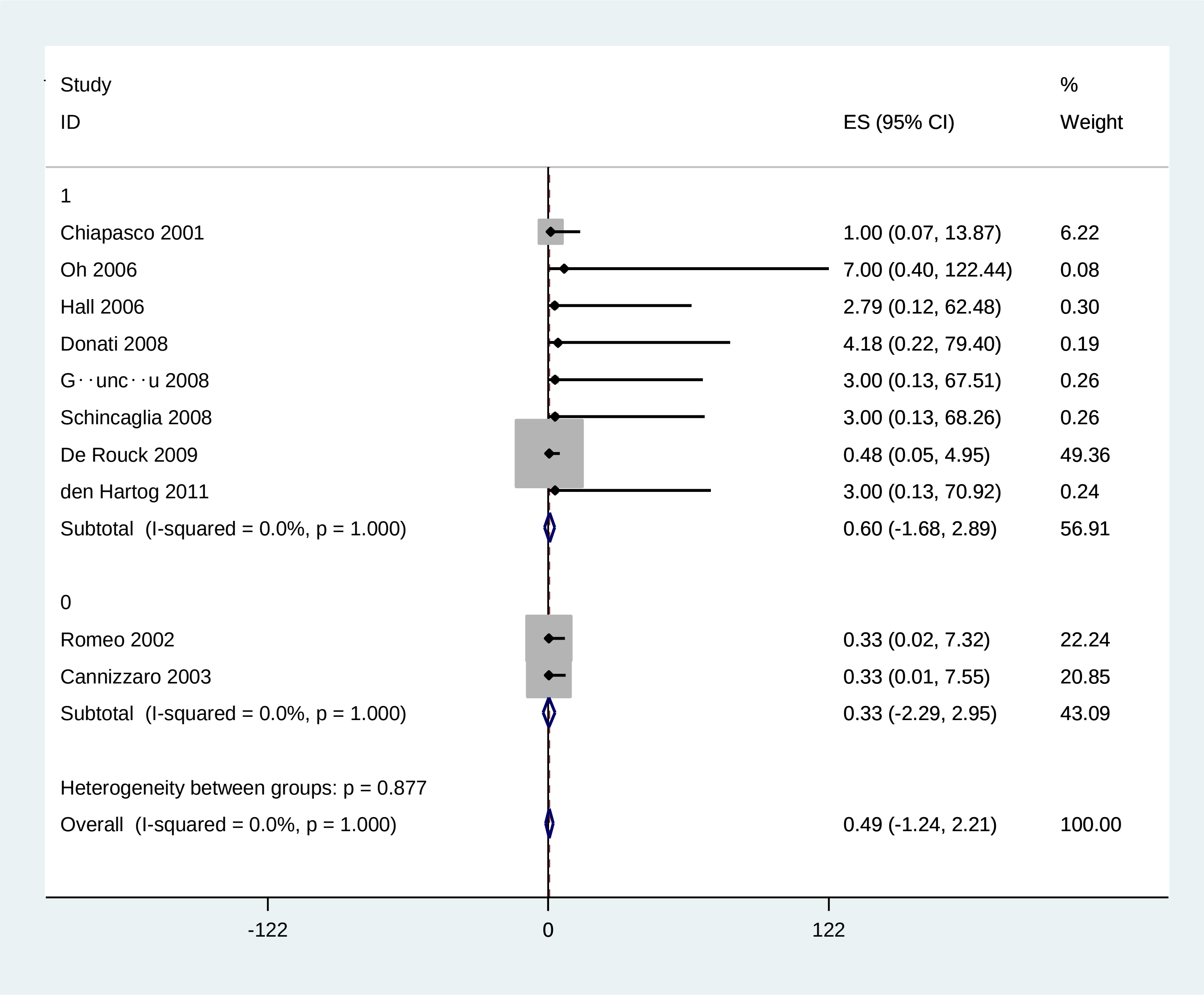
Study
ID
ES (95% CI)
%
Weight
1
Chiapasco 2001 1.00 (0.07, 13.87) 6.22
Oh 2006 7.00 (0.40, 122.44) 0.08
Hall 2006 2.79 (0.12, 62.48) 0.30
Donati 2008 4.18 (0.22, 79.40) 0.19
G··unc··u 2008 3.00 (0.13, 67.51) 0.26
Schincaglia 2008 3.00 (0.13, 68.26) 0.26
De Rouck 2009 0.48 (0.05, 4.95) 49.36
den Hartog 2011 3.00 (0.13, 70.92) 0.24
Subtotal (I-squared = 0.0%, p = 1.000) 0.60 (-1.68, 2.89) 56.91
0
Romeo 2002 0.33 (0.02, 7.32) 22.24
Cannizzaro 2003 0.33 (0.01, 7.55) 20.85
Subtotal (I-squared = 0.0%, p = 1.000) 0.33 (-2.29, 2.95) 43.09
Heterogeneity between groups: p = 0.877
Overall (I-squared = 0.0%, p = 1.000) 0.49 (-1.24, 2.21) 100.00
-122
0
122
