# Supplementary material for: Effect of Risk of Bias on the Effect Size of Meta-Analytic Estimates in Randomized Controlled Trials in Periodontology and Implant Dentistry
Source: PLoS One. 2015 Sep 30;10(9):e0139030. doi: 10.1371/journal.pone.0139030 (PMC4589402; doi:10.1371/journal.pone.0139030)
Supplement: S1 Table — (DOCX) [file pone.0139030.s003.docx]

**Supporting Information**

S1 Table: Risk of bias summary of RCTs included in Esposito et al. 2013

| **Study** | **Random sequence generation** | **Allocation concealment** | **Blinding of outcome assessment** | **Incomplete outcome data** | **Selective reporting** | **Other bias** |
| --- | --- | --- | --- | --- | --- | --- |
| Assad 2007 | Unclear | Unclear | Unclear | Low | Low | Low |
| Cannizzaro 2003 | Low | High | Low | Low | Low | Low |
| Cannizzaro 2008a | Low | Low | Low | Low | Low | Low |
| Cannizzaro 2008b | Low | Low | Low | Low | Low | Unclear |
| Cannizzaro 2008d | Low | Low | Low | Low | Low | Low |
| Cannizzaro 2010 | Low | Low | Unclear | Low | Low | Low |
| Chiapasco 2001 | High | High | Low | Low | Low | Low |
| Crespi 2008 | Unclear | Unclear | Unclear | Low | Low | Low |
| den hartog 2011 | Low | Low | High | Low | Low | Low |
| De Rouck 2009 | Low | Unclear | Unclear | Low | Low | High |
| Donati 2008 | Low | Unclear | Unclear | Low | Low | Low |
| Enkling 2010 | Unclear | Unclear | Low | Low | Low | Low |
| Fischer 2004 | Unclear | High | High | Low | Low | Low |
| Güncü 2008 | Unclear | High | Low | Low | Low | Low |
| Hall 2006 | Unclear | Unclear | Low | Low | Low | Low |
| Lindeboom 2006 | Low | Low | Low | Low | Low | Unclear |
| Meloni 2012 | Low | Low | Unclear | Low | Low | Low |
| Merli 2008 | Low | Low | Unclear | Low | Low | High |
| Oh 2006 | Unclear | Unclear | Low | Low | Low | Low |
| Payne 2002 | Low | Unclear | Low | Low | Low | Low |
| Romeo 2002 | Low | High | Low | Low | Low | Low |
| Schincaglia 2008 | Low | High | Unclear | Low | Low | Low |
| Tawse-Smith 2002 | Low | Unclear | Low | Low | Low | High |
| Testori 2007 | Low | Low | Unclear | Low | Low | Unclear |
| Turkyilmaz 2007 | Unclear | High | High | Low | Low | Low |
| Zöllner 2008 | Low | High | Unclear | High | Low | Low |
