# Supplementary material for: Effect of Risk of Bias on the Effect Size of Meta-Analytic Estimates in Randomized Controlled Trials in Periodontology and Implant Dentistry
Source: PLoS One. 2015 Sep 30;10(9):e0139030. doi: 10.1371/journal.pone.0139030 (PMC4589402; doi:10.1371/journal.pone.0139030)
Supplement: S2 Table — (DOCX) [file pone.0139030.s004.docx]

**Supporting Information**

S2 Table: Risk of bias summary of RCTs included in Riley and Lamont 2013

| **Study** | **Random sequence generation** | **Allocation concealment** | **Bliniding of particpants** | **Blinding of outcome assessment** | **Incomplete outcome data** | **Selective reporting** | **Other bias** |
| --- | --- | --- | --- | --- | --- | --- | --- |
| Allen 2002 | Low | Low | Low | Low | Low | High | Low |
| Bolden 1992 | Low | Low | Low | Low | Low | Low | Low |
| Cubbels 1991 | Low | Low | Low | Low | Low | Low | Low |
| Deasy 1991 | Low | Low | Low | Low | Unclear | Low | Low |
| Denepitiya 1992 | Low | Low | Low | Low | Low | Low | Low |
| Ellwood 1998 | Low | Low | Low | Low | High | High | Low |
| Feller 1996 | Low | Low | Low | Low | Low | Low | Low |
| Garcia-Godoy 1990 | Low | Low | Low | Low | Low | Low | Low |
| Hawley 1995 | Low | Low | Low | Low | unclear | Low | Low |
| Hu 1997 | Low | Low | Low | Low | Low | Low | Low |
| Kanchanakamol 1995 | Low | Low | High | Low | Unclear | Low | Low |
| Kraivaphan 2006 | Unclear | Unclear | Low | Low | Unclear | Low | Unclear |
| Lindhe 1993 | Low | Low | Low | Low | Low | High | Low |
| Liu 2002 | Unclear | Unclear | Low | Low | Low | High | Low |
| Lobene 1991 | Low | Low | Low | Low | Unclear | Low | Low |
| Mankodi 1992 | Low | Low | Low | Low | Low | Low | Low |
| Mankodi 2011 | Low | Low | Low | Low | Low | Low | Unclear |
| Mann 1996 | Low | Low | Low | Low | Unclear | Low | Low |
| Mann 2001 | Low | Low | Low | Low | Unclear | Low | Low |
| Mateu 2008 | Low | Low | Low | Low | Unclear | Low | Low |
| McClanahan 1997 | Low | Unclear | Low | Low | High | Low | Unclear |
| Palomo 1994 | Low | Low | Low | Low | Low | High | Low |
| Pradeep 2012 | Low | Low | Low | Low | Low | High | Unclear |
| Renvert 1995 | Unclear | Unclear | Low | Low | Low | Low | Unclear |
| Schiff 2006 | Low | Low | Low | Low | Low | Low | Low |
| Svatun 1993 | Unclear | Unclear | Low | Low | Unclear | Low | Unclear |
| Triratana 1993 | Low | Low | Low | Low | Low | Low | Low |
| Triratana 1994 | Unclear | Unclear | Low | Low | Low | Low | Unclear |
| Triratana 2002 | Low | Low | Low | Low | Low | Low | Low |
| Vered 2009 | Low | Low | Low | Low | Unclear | High | Low |
