# Supplementary material for: Effect of Risk of Bias on the Effect Size of Meta-Analytic Estimates in Randomized Controlled Trials in Periodontology and Implant Dentistry
Source: PLoS One. 2015 Sep 30;10(9):e0139030. doi: 10.1371/journal.pone.0139030 (PMC4589402; doi:10.1371/journal.pone.0139030)
Supplement: S3 Table — (DOCX) [file pone.0139030.s005.docx]

S3: Risk of bias summary of RCTs included in Yaacob et al. 2013

| **Study** | **Random sequence generation** | **Allocation concealment** | **Blinding of outcome assessment** | **Incomplete outcome data** | **Selective reporting** | **Other bias** |
| --- | --- | --- | --- | --- | --- | --- |
| Ainamo 1997 | Unclear | Unclear | Low | Low | Low | Low |
| Baab 1989 | Unclear | Low | Low | Low | Low | Low |
| Barnes 1993 | Unclear | Unclear | Low | Low | Low | Low |
| Biavati Silvestrini 2010 | Unclear | Unclear | Unclear | Low | Low | Low |
| Biesbrock 2007 | Unclear | Low | Low | Low | Low | Low |
| Clerehugh 1988 | Low | Low | Low | Low | Low | Low |
| Costa 2007 | Unclear | Unclear | Unclear | Low | Low | Low |
| Cronin 1998 | Low | Unclear | Low | Low | Low | Low |
| Dentino 2002 | Low | Unclear | Low | Unclear | Low | Low |
| Dorfer 2009 | Unclear | Unclear | Low | Low | Low | Low |
| Emling 1991 | Unclear | Unclear | Low | Low | Low | Low |
| Forgas-B 1998 | Unclear | Unclear | Low | Unclear | Low | Low |
| Galgut 1996 | Low | Low | Low | Unclear | Low | Low |
| Garcia-Godoy 2001 | Unclear | Unclear | Low | Low | Low | Low |
| Glass 1965 | Low | Low | Low | High | Low | Low |
| Goyal 2007 | Unclear | Unclear | Low | Low | Low | Low |
| Gugerli 2007 | Low | Unclear | Low | Low | Low | Low |
| Haffajee 2001a | Low | Low | Low | Low | Low | Low |
| Heasman 1999 | Unclear | Unclear | Low | Low | Low | Low |
| Hickman 2002 | Low | Unclear | Low | Low | Low | Low |
| Ho 1997 | Low | Unclear | Low | Unclear | Low | Low |
| Johnson 1994 | Unclear | Unclear | Low | Low | Low | Low |
| Kallar 2011 | Unclear | Unclear | Unclear | Unclear | High | Unclear |
| Khocht 1992 | Unclear | Unclear | Low | Low | Low | Low |
| Lapiere unpublished | Unclear | Unclear | Low | Low | Low | Low |
| Lazarescu 2003 | High | Unclear | Low | Low | Low | Low |
| Lobene 1964a | Unclear | Unclear | Low | Unclear | Low | Low |
| McCracken 2004 | Low | Unclear | Low | Low | Low | Low |
| McCracken 2009 | Low | Low | Low | Low | Low | Low |
| Moreira 2007 | Low | Unclear | Low | Low | Low | Low |
| Moritis 2008 | Unclear | Unclear | Low | Low | Low | Low |
| O´Beirne 1996 | Unclear | Low | Low | Low | Low | Low |
| Pucher 1999 | Unclear | Low | Low | Unclear | Low | Low |
| Rosema 2008 | Low | Unclear | Low | Low | Low | Low |
| Sharma 2000 | Unclear | Unclear | Unclear | Low | Low | Low |
| Sharma 2010 | Low | Low | Low | Low | Low | Low |
| Silverman 2004 | Low | Low | Low | Low | Low | Low |
| Singh unpublished | Unclear | Unclear | Unclear | Unclear | Low | Low |
| Soparkar 1964 | Unclear | Unclear | Unclear | Unclear | Low | Low |
| Soparkar 2000 | Unclear | Unclear | Low | Low | Low | Low |
| Sowinski 2000 | Unclear | Unclear | Low | Low | Low | Low |
| Stabholz 1996 | Unclear | Unclear | Low | Low | Low | Low |
| Stoltze 1994 | Unclear | Unclear | Unclear | Low | Low | Low |
| Terezhalmy 1995a | Unclear | Unclear | Unclear | Low | Low | Low |
| Toto 1966 | Unclear | Unclear | Unclear | Low | Low | Low |
| Tritten 1996 | Low | Unclear | Low | Low | Low | Low |
| Van der Weijden 1994 | Unclear | Unclear | Low | Low | Low | Low |
| Van Swol 1996 | Low | Low | Low | Unclear | Low | Low |
| Walsh 1989 | High | Unclear | Low | Unclear | Low | Low |
| Warren 2001 | Low | Unclear | Low | Low | Low | Low |
| Wilson 1993 | Unclear | Unclear | Low | High | Low | Low |
| Yankell 1996 | Unclear | Unclear | Low | Unclear | Low | Low |
| Yankel 1997 | Unclear | Unclear | Low | Unclear | Low | Low |
| Yukna 1993b | Unclear | Unclear | Low | Low | Low | Unclear |
| Zimmer 2002 | Unclear | Low | Low | Low | Low | Low |
| Zimmer 2005 | Unclear | Unclear | Low | Low | Low | Low |
